# pHLIP ICG for delineation of tumors and blood flow during fluorescence-guided surgery

**DOI:** 10.1038/s41598-020-75443-5

**Published:** 2020-10-27

**Authors:** Troy Crawford, Anna Moshnikova, Sean Roles, Dhammika Weerakkody, Michael DuPont, Lukas M. Carter, John Shen, Donald M. Engelman, Jason S. Lewis, Oleg A. Andreev, Yana K. Reshetnyak

**Affiliations:** 1 Physics Department, University of Rhode Island, Kingston, RI USA; 2 Department of Radiology and the Program in Molecular Pharmacology, Memorial Sloan Kettering Cancer Center, New York, NY USA; 3 Stryker Endoscopy Corp., 5900 Optical Ct, San Jose, CA 95138 USA; 4 Department of Molecular Biophysics and Biochemistry, Yale University, New Haven, CT USA

**Keywords:** Translational research, Surgical oncology

## Abstract

Fluorescence imaging has seen enduring use in blood flow visualization and is now finding a new range of applications in image-guided surgery. In this paper, we report a translational study of a new fluorescent agent for use in surgery, pHLIP ICG, where ICG (indocyanine green) is a surgical fluorescent dye used widely for imaging blood flow. We studied pHLIP ICG interaction with the cell membrane lipid bilayer, the pharmacology and toxicology in vitro and in vivo (mice and dogs), and the biodistribution and clearance of pHLIP ICG in mice. The pHLIP ICG tumor targeting and imaging efficacy studies were carried out in several murine and human mouse tumor models. Blood vessels were imaged in mice and pigs. Clinical Stryker imaging instruments for endoscopy and open surgery were used in the study. Intravenously administered pHLIP ICG exhibits a multi-hour circulation half-life, offering protracted delineation of vasculature. As it clears from the blood, pHLIP ICG targets tumors and tumor stroma, marking them for surgical removal. pHLIP ICG is non-toxic, marks blood flow for hours after injection, and effectively delineates tumors for improved resection on the day after administration.

## Introduction

**Figure 1:**
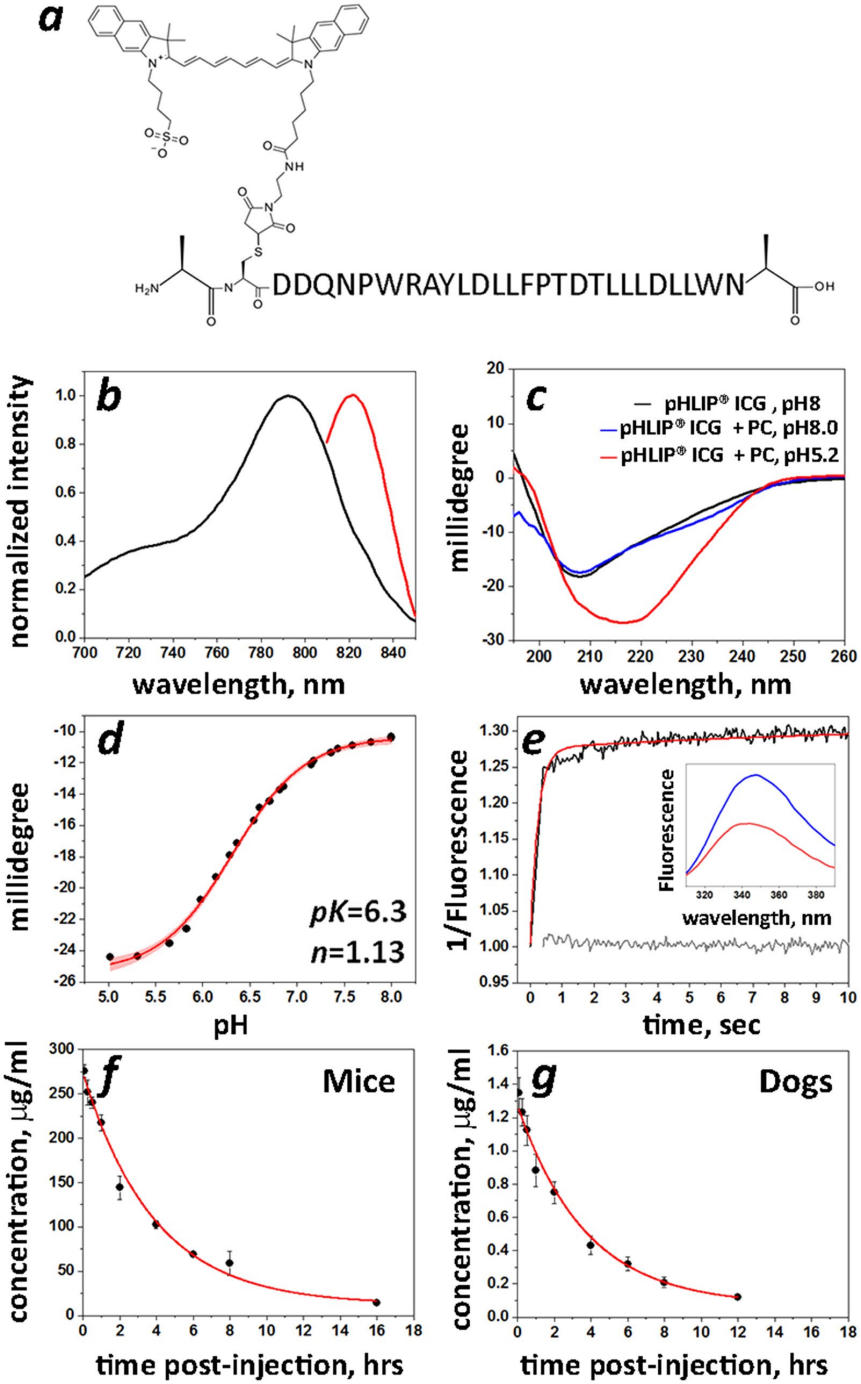
Characterization of pHLIP ICG and blood clearance in mice and dogs. (**a**) The Var3 pHLIP sequence is shown with the chemical structure of the ICG dye coupled to a single Cys residue at the N-terminal part of pHLIP peptide. (**b**) Normalized absorbance (black line) and fluorescence (red line) spectra (the excitation is at 805 nm) of pHLIP ICG (5 µM) measured in DMF. (**c**) Circular dichroism (CD) spectra of pHLIP ICG measured in phosphate buffer (pH 8) in absence (pHLIP ICG, pH 8—black line) and presence (pHLIP ICG + PC, pH 8—blue line) of POPC liposomes, and in the presence of POPC liposomes at pH 5.2 (pHLIP ICG + PC, pH 5.2—red line) indicate on formation of helical structure in membrane at low pH. (**d**) pH-dependent insertion of pHLIP ICG into the bilayers of POPC liposomes monitored by changes of the CD spectral signal at 222 nm. The data were fitted with the Henderson-Hasselbach equation (red line with 95% confidence interval) to establish the midpoint of the transition (pK). (**e**) The kinetics signal (multiplied by − 1) of pHLIP ICG insertion into lipid bilayers of POPC liposomes as a result of a pH drop from pH 8 (blue line in insert) to pH 5 (red line in insert) was monitored in real-time by the tryptophan fluorescence changes (black line). The gray line is a baseline obtained by mixing pHLIP ICG with POPC liposomes at pH 8 with pH 8 buffer. The red line is a bi-exponential fit of the experimental data (85% of the signal changes occur with a rate of 5 s^−1^ and about 15% of final adjustment is completed at a rate of 0.077 s^−1^). (**f**,**g**) Blood clearance profile and single exponential fitting curves (red lines) obtained from mice (**f**) and dogs (**g**) after a single i.v. injection of 12.3 mg/kg of pHLIP ICG to mice and 0.064 mg/kg of pHLIP ICG to dogs are shown.

More than a decade ago, the first pH-Low Insertion Peptide (pHLIP) was introduced as an agent for targeting acidity at the surfaces of cellular membranes in vitro and in vivo^1,2^. The molecular mechanism of action of acidity targeting by peptides of the pHLIP family has been well-investigated and has been the subject of many publications (see review^3^ and references within it). pHLIP is a water-soluble, moderately hydrophobic peptide containing several carboxyl groups in a sequence of hydrophobic and polar residues (Fig. 1a). The dielectric environment at a membrane shifts the p*K*a’s of these carboxyl groups toward higher pHs, and a moderately low local pH (pH 5–7) promotes the protonation of Asp and Glu residues and the C-terminal, membrane-inserting end of pHLIP peptide. Protonation increases the peptide’s hydrophobicity and triggers its partition into a membrane, which is accompanied by a coil-helix transition to form a stable helix across the membrane (transmembrane helix). pHLIP peptides target tumors with high precision, since high cell surface acidity or low pH (pH_surf_) is a byproduct of the elevated glycolytic metabolism and of overactivated carbonic anhydrases (CAIX) at cancer and activated immune cells^4–7^. A variety of imaging and therapeutic agents are successfully delivered to tumors by pHLIP peptides (see review^8^ and references within it). pHLIP peptides tumor targeting has been demonstrated in human cancerous tissues and more than 20 different human and murine cancer models including transgenic breast, prostate, pancreatic and skin models. pHLIP peptides tumor uptake was correlated with tumor extracellular pH^9–11^. pHLIP peptides tumor targeting has been shown to be enhanced in tumors that are further acidified by co-injection of glucose^12^ or by overexpression of CAIX at the surface of HCT116 cancer cells^10^. Alternatively, pHLIP peptides tumor targeting was shown to be reduced by alkalization of tumors in mice fed with bicarbonate drinking water^13^. Micro-metastases near primary tumors and in distant organs were shown to be very well targeted by pHLIP peptides^12,14–16^. In multiple studies, it has been found that non-protonatable (at low pH) pHLIP variants, where some or all Asp/Glu residues were replaced by Lys, did not target tumors and did not exhibit pH-dependent membrane insertion. pHLIP peptides distribution within tumors, cellular localization and correlation with a variety of markers have been intensively investigated^12,13,17,18^.

Since pHLIP peptides offer a unique approach to the targeting of a spectrum of tumor subtypes, we were motivated to develop a fluorescent pHLIP candidate in order to improve the surgical resection of tumors guided by fluorescence. After testing a variety of fluorescent dyes with pHLIP peptides^14^, we chose a widely used clinical fluorescent dye, indocyanine green (ICG). In addition to its long record of safety, ICG was chosen to couple to pHLIP peptide as a clinical imaging candidate for two main reasons: first, the fluorescence of pHLIP ICG is enhanced about 20 times when pHLIP tethers ICG to the membrane, as compared to the emission of pHLIP ICG in aqueous solution^19,20^, thus reducing the probability of false positive signal and contrast-to-noise ratio^12^. Second, many clinical instruments for imaging ICG fluorescence have already been developed and are clinically available^21^, which streamlines pHLIP ICG’s clinical adoption. Here we present the results of the translational studies, including in vitro and in vivo efficacy, pharmacology and toxicology, that reveal important properties of a pHLIP agent and form the basis for the clinical trials that will soon be initiated.

## Methods

All manufacturing procedures were completed in accordance with good laboratory practice (GLP) quality control specifications. pHLIP ICG was synthesized and the agent was produced by Iris Biotech, GmbH (Germany) in partnership with Chemical and Biopharmaceutical Laboratories (CBL) (Greece) and then further optimized by CordenPharma. GLP pHLIP ICG verification batch #1912127 was manufactured in Frankfurt by CordenPharma; the acceptance criteria are presented in Supplementary Table SI,1. Stability studies were completed with pHLIP ICG formulations in PBS and PBS containing 5% DMSO or 5% Ethanol (vol/vol). Biophysical studies were carried out using POPC (1-palmitoyl-2-oleoyl-sn-glycero-3-phosphocholine) liposomes as mimics of cell membrane bilayers. Cytotoxicity was completed on human mammary epithelial cells (HMEpC), which were acquired from Cell Applications, Inc. and were authenticated and stored according to supplier’s instructions. Hemolysis assays were carried out using single donor human whole blood purchased from Innovative Research, Inc. The pHLIP ICG enzyme binding assays were performed by Eurofins Panlabs, Inc. Murine animal studies were conducted at the University of Rhode Island (URI) according to the approved by URI Institution Animal Care and Use Committee (IACUC) animal protocol AN04-12-011. Animal studies in pigs were conducted at the Porcine Laboratory, Sutter Institute for Medical Research (SIMR) according to the approved by SIMR IACUC animal protocol STE.10.19 (Stryker Endoscopy Imaging and Instrumentation Studies). The studies complied with the principles and procedures outlined by the National Institutes of Health for the care and use of animals. Animal studies at the Memorial Sloan Kettering Cancer Center (MSK) Antitumor Assessment Core Facility and Charles River Labs were conducted in accordance with approved Study Protocols and in compliance with the Department of Health and Human Services, Food and Drug Administration (FDA), United States Code of Federal Regulations, Title 21, Pad: 58: Good Laboratory Practice for Nonclinical Laboratory Studies and as accepted by Regulatory Authorities throughout the European Union (OECD Principles of Good Laboratory Practice), and other countries that are signatories to the OECD Mutual Acceptance of Data Agreement. Detailed descriptions of all methods are presented in the Supporting Information.

## Results

### pHLIP ICG synthesis and characterization

The 28-amino-acid pHLIP Var3 peptide with free N- and C-terminus was synthesized on solid support followed by purification and conjugation of its single Cys residue with ICG-maleimide to obtain the pHLIP ICG imaging agent (Fig. 1a, Supplementary Figure SI,1a). Further optimization of the pHLIP ICG manufacturing protocol has been completed by CordenPharma. pHLIP ICG reconstituted in PBS was found to be stable in solution for 4 days at room temperature.

The molar attenuation coefficient (i.e. extinction coefficient) of pHLIP ICG at 810 nm measured in methanol (Supplementary Figure SI,2a) was determined to be 153,000 M^−1^ cm^−1^ and was used to calculate the concentration of pHLIP ICG. The absorption and emission spectra of pHLIP ICG in DMF (to mimic the hydrophobic environment of the membrane) are shown in Fig. 1b. It is important to note that spectral signals of pHLIP ICG do not overlap with the absorption or emission of Methylene Blue and Isosulfan Blue, dyes which are widely used for lymph nodes staining during surgery (Supplementary Figure SI,2b–f). The interaction of pHLIP ICG with membrane lipid bilayers was studied using POPC liposomes. pHLIP ICG exhibits pHLIP-like pH-dependent interactions with cell membranes monitored by changes of the peptide circular dichroism and the fluorescence emission of tryptophan residues (Fig. 1c–e). pHLIP ICG is inserted into lipid bilayer of membrane and forms alpha helical structure at low pHs, as do all peptides of pHLIP family^3,22^. The midpoint of the transition for pHLIP ICG insertion in liposomes is at pH 6.3 as calculated from graph presented on Fig. 1d. Thus, at pH 6.0 (below of midpoint), pH which is found at the surface of cancer cells within tumors^23,24^, about 70% of pHLIP ICG molecules are expected to be inserted across plasma membrane at equilibrium, while at pH 7.4 (the surface pH of normal cells) < 1% of pHLIP ICG molecules are expected to be inserted into the membranes. Since the rate constant of pHLIP ICG insertion into a membrane is high (approximately 5 s^−1^), the insertion will occur even under conditions of fast blood flow and limited exposure time of pHLIP ICG to acidic cancer cells.

### Safety studies in vitro

Safety pharmacology profiling of pHLIP ICG (2 µM) was performed in vitro using 86 enzyme and receptor targets. No inhibitory effect was observed, except a mild inhibitory effect of pHLIP ICG on progesterone receptor B: the established *IC*_*50*_ and *K*_*i*_ for pHLIP ICG are 0.64 µM and 0.51 µM, respectively (for comparison, the *IC*_*50*_ and *K*_*i*_ for the known R-2050 progesterone receptor agonist are several-fold lower: 0.33 nM and 0.26 nM, respectively; Supplementary Figure SI,3). It is unlikely that a significant amount of pHLIP ICG would reach the nucleus after a single i.v. administration of the agent, since it binds to cellular membranes.

The cytotoxicity of pHLIP ICG was assessed by treatment of human mammary epithelial cells (HMEpC) with increasing concentrations of pHLIP ICG (up to 16 µM) for 72 h, followed by assessment of cell viability. pHLIP ICG did not show any cytotoxic effect at any tested concentration.

Hemolysis assays were performed on single donor human whole blood samples treated with increasing concentrations (up to 8 nmol/mL) of pHLIP ICG. Hemolysis was assessed by the release of hemoglobin into the supernatant as monitored by measuring absorbance at 450 nm after centrifugation to remove red blood cells (RBC). The amount of RBC lysis was less than 2% in all samples compared to 100% release upon lysis by water or Triton X-100.

The genotoxicity of pHLIP ICG was evaluated using in vitro bacterial reverse mutation and mammalian cell micronucleus tests in human peripheral blood lymphocytes (see details in Supplementary Information). pHLIP ICG did not show any evidence of genotoxic activity in these in vitro mutagenicity assays.

### Pharmacology

Pharmacokinetic studies were performed in mice and dogs. Blood samples were collected at 5, 15, 30 min and 1, 2, 4, 6, 8, 12 or 16, 24 and 48 h after a single i.v. dose of pHLIP ICG (12.3 mg/kg to mice and 0.064 mg/kg to dogs). Evidence of systemic exposure to pHLIP ICG was observed in all animals and was quantifiable up to 8–16 h post administration (Supplementary Table SI,2). Single exponential decays were observed in both cases (Fig. 1f,g). The mean clearance (*Cl*), volume of distribution (*V*_*z*_) and elimination half-time (*T*_*1/2*_) values obtained for mice and dogs were very similar: *Cl* = 9.53 mL/h/kg (mice) and 11.6 mL/h/kg (dogs); *V*_z_ = 53.51 mL/kg (mice) and 61.6 mL/kg (dogs); and *T*_*1/*2_ = 3.9 h (mice) and 3.7 h (dogs).

### Blood vessel imaging

**Figure 2:**
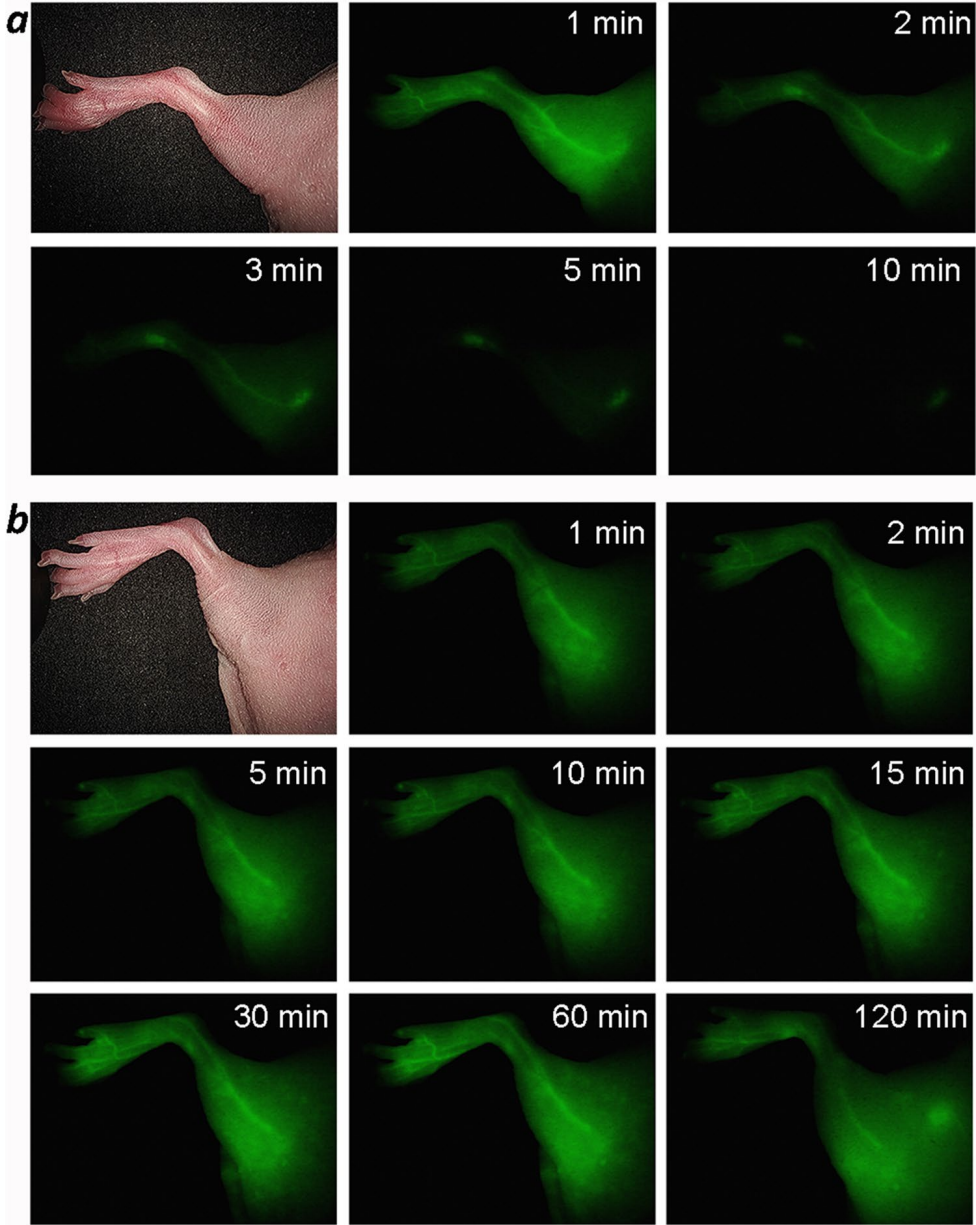
Imaging of blood vessels in mice. Representative NIR pHLIP ICG fluorescent images of blood vessels at different time points after a single i.v. injection of ICG-Cys (**a**) or pHLIP ICG (**b**).

**Figure 3:**
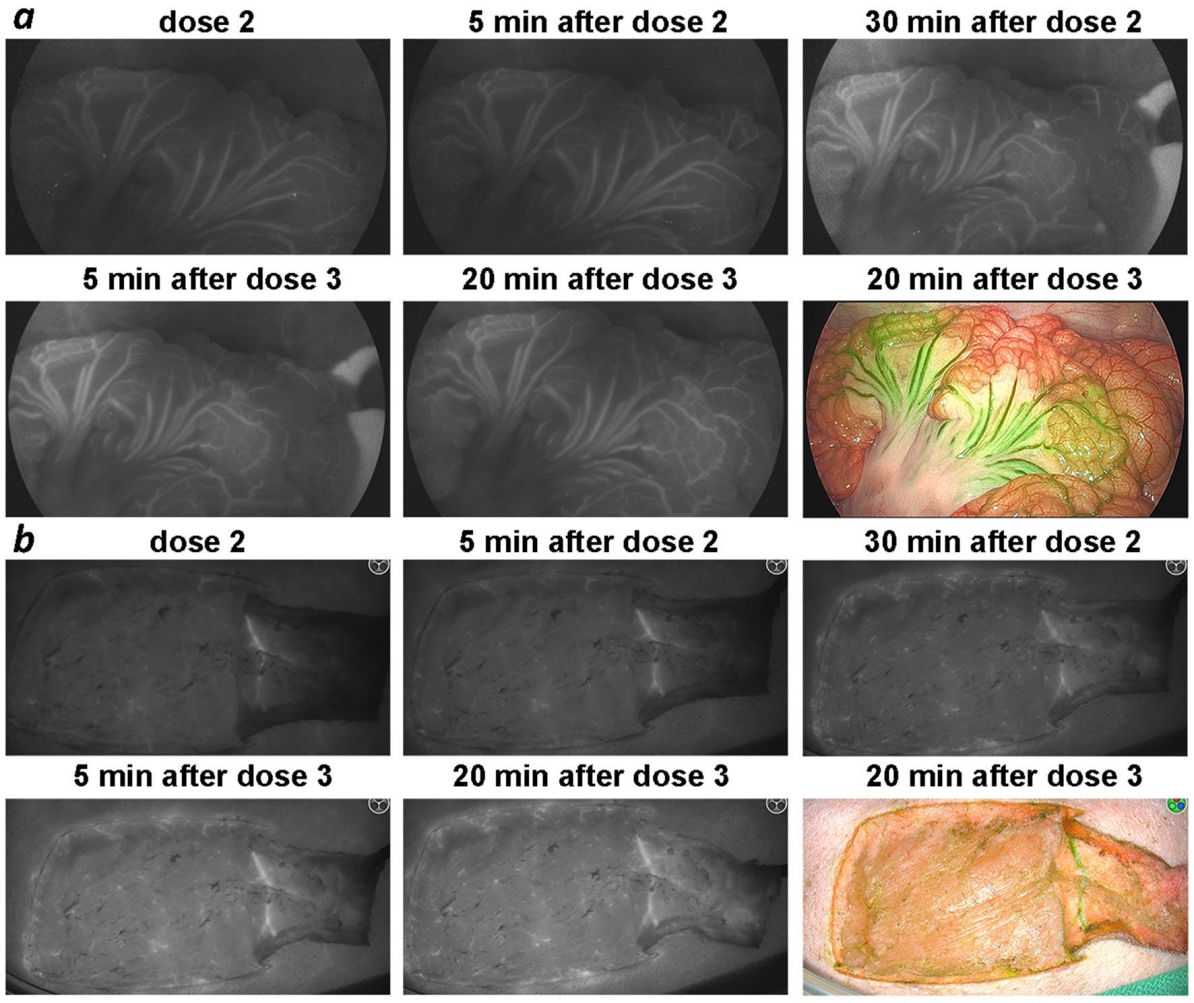
Imaging of blood vessels in pigs. Representative NIR pHLIP ICG (verification batch #1912127) fluorescent images and overlay of fluorescent and color images of blood vessels at different time points after injection of pHLIP ICG in PBS (dose 1—0.052 mg/kg; dose 2—additional 0.12 mg/kg; dose 3—additional 0.24 mg/kg) are shown. (**a**) Imaging inside the pig body cavity was performed using a Stryker 1688 AIM system (808 nm excitation) for endoscopic/laparoscopic imaging (gain 8/10 for dose 2, gain 7/10 for dose 3). (**b**) Imaging of a pedicle flap was performed using a Stryker SPY-PHI handheld device (805 nm excitation) for open-field imaging.

Our pharmacology data show that pHLIP ICG remains at significant levels in the blood for several hours—an essential feature of good blood pool agents for fluorescence angiography. ICG, when used as a contrast agent for imaging blood flow, is cleared from the blood within a few minutes, limiting the time window for imaging. We carried out a comparative study of blood flow imaging in mice using ICG-Cys (ICG-maleimide conjugated with a Cys residue) and pHLIP ICG (2.5 nmol of each). As expected, ICG-Cys was cleared from the blood within the first 5 min (Fig. 2a), whereas pHLIP ICG clearly revealed the blood vessels for up to 2 h (Fig. 2b and Supplementary Figure SI,4). To further investigate the potential utility of pHLIP ICG in fluorescence angiography, blood perfusion was imaged in pigs, since the blood circulation of pigs is closely related to that of humans. Pigs received several doses of pHLIP ICG: 0.052 mg/kg (dose 1) followed by additional 0.12 mg/kg (dose 2) and then an additional 0.24 mg/kg (dose 3). Visualization of pHLIP ICG NIR fluorescence in blood vessels was performed using two systems: (i) for endoscopic/laparoscopic imaging inside the pig body cavity (Fig. 3a, Supplementary Figure SI,5a and Supplementary Video SI,1), and (ii) open-field imaging of a pedicle flap (Fig. 3b, Supplementary Figure SI,5b and Supplementary Video SI,2). Illuminated blood vessels were clearly visible at 2 h post-injection (Supplementary Figure SI,5), establishing the superiority of pHLIP ICG use over ICG in fluorescence angiography and other blood flow/perfusion applications, when prolonged imaging is required.

### Biodistribution and tumor targeting

**Figure 4:**
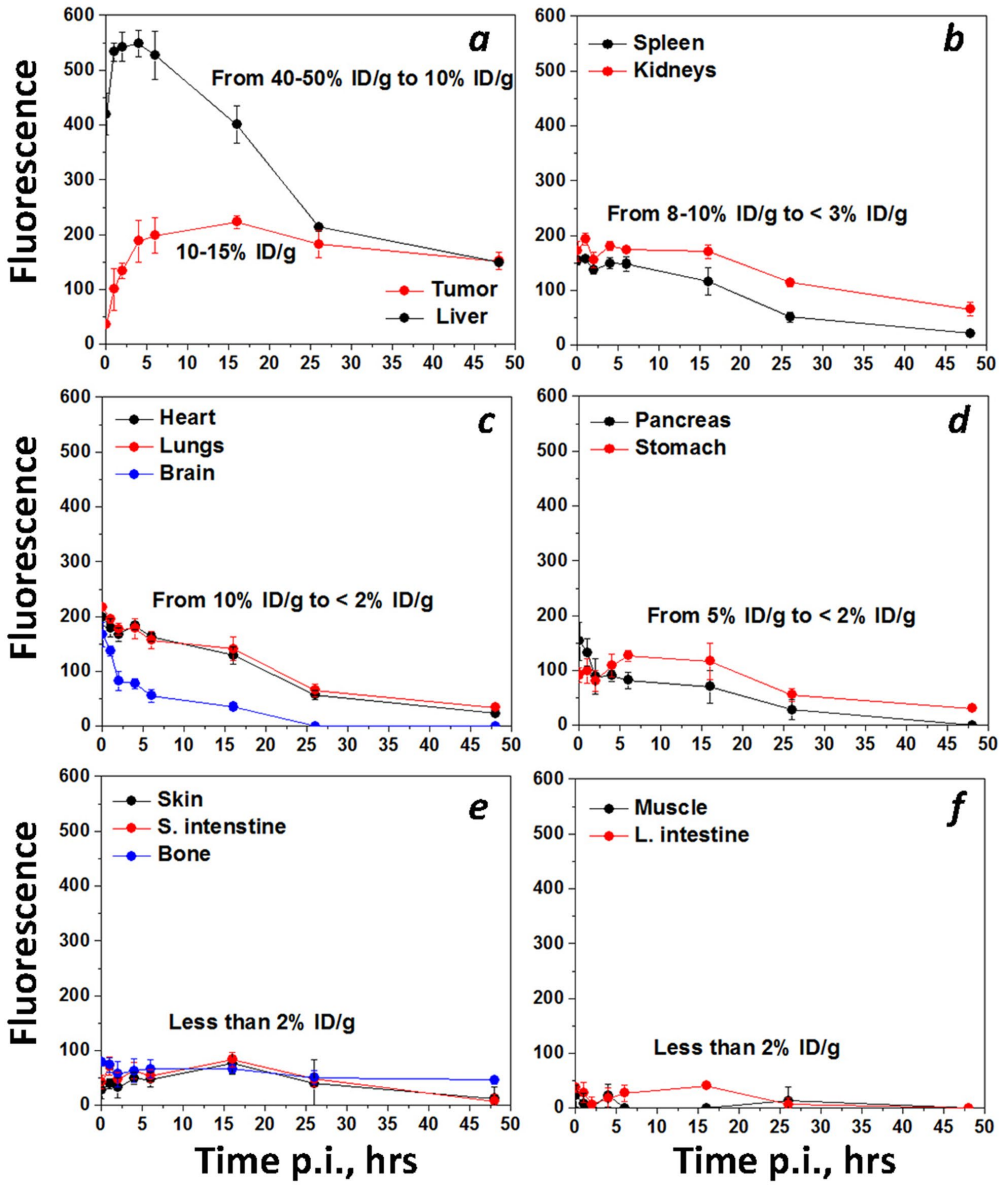
Kinetics. The calculated mean and standard deviation values at different time points post-dose are shown. Values are given in Supplementary Table SI,5. The description of %ID/g calculations is presented in the main text and “Methods” section.

Biodistribution and tumor targeting were assessed after a single tail vein injection of pHLIP ICG in mice bearing murine and human tumors. Biodistribution studies were carried out in female and male mice bearing a triple-negative 4T1 tumor, which closely mimics stage IV human breast cancer. Targeting of 4T1 tumors in BALB/c mice by pHLIP ICG is shown in Supplementary Figure SI,6. To establish the biodistribution of pHLIP ICG, animals were euthanized at different time points (5 min, 1, 2, 4, 6, 16, 26 and 48 h) and organs were collected and imaged. Representative images of organs are shown in Supplementary Figure SI,7; values of fluorescence intensity are shown in Supplementary Table SI,3 and mean values are given in Supplementary Table SI,4; the calculated signal level in each organ at different time points is shown in Supplementary Figure SI,8. The kinetics of the fluorescence signal changes in organs and tissue is shown in Fig. 4. Organs and tissues were frozen after imaging and processed to measure the signal in the tissue/organ homogenates. The measurement precision of the amount of fluorescent compound in the tissues and organs is limited, but estimates are possible. The estimations were made from a calibration curve obtained by spiking known amounts of pHLIP ICG with organs and tissue homogenates collected from control animals. According to our estimates, the amount of pHLIP ICG within the tumor reaches about 10–15% ID/g at 4 h post injection and stays constant up to 24 h, decaying slightly at 48 h. The clearance was observed to be predominantly hepatic, and the level of the signal increases in the liver with time and reaches a maximum level at 1-h post-administration, followed by signal decay after 6 h. Well-perfused organs like the heart, kidney and lungs were imaged (no perfusion with buffer); it is evident that a significant amount of the signal (especially at earlier time points) comes from the blood associated with these organs, and that the signal level decays with blood clearance. Another group of organs, including the spleen, pancreas, stomach and brain, has a lower signal level, which also decays with blood clearance. Small and large intestines, bone, skin and muscle (and prostate in male animals, not shown here) have very low signal levels, estimated to be less than 2% ID/g at all time points. At 20–48 h, when the blood has cleared and the fluorescence signal in all organs is minimal, the contrast between the tumor and the surrounding tissue or healthy organs is significant.

**Figure 5:**
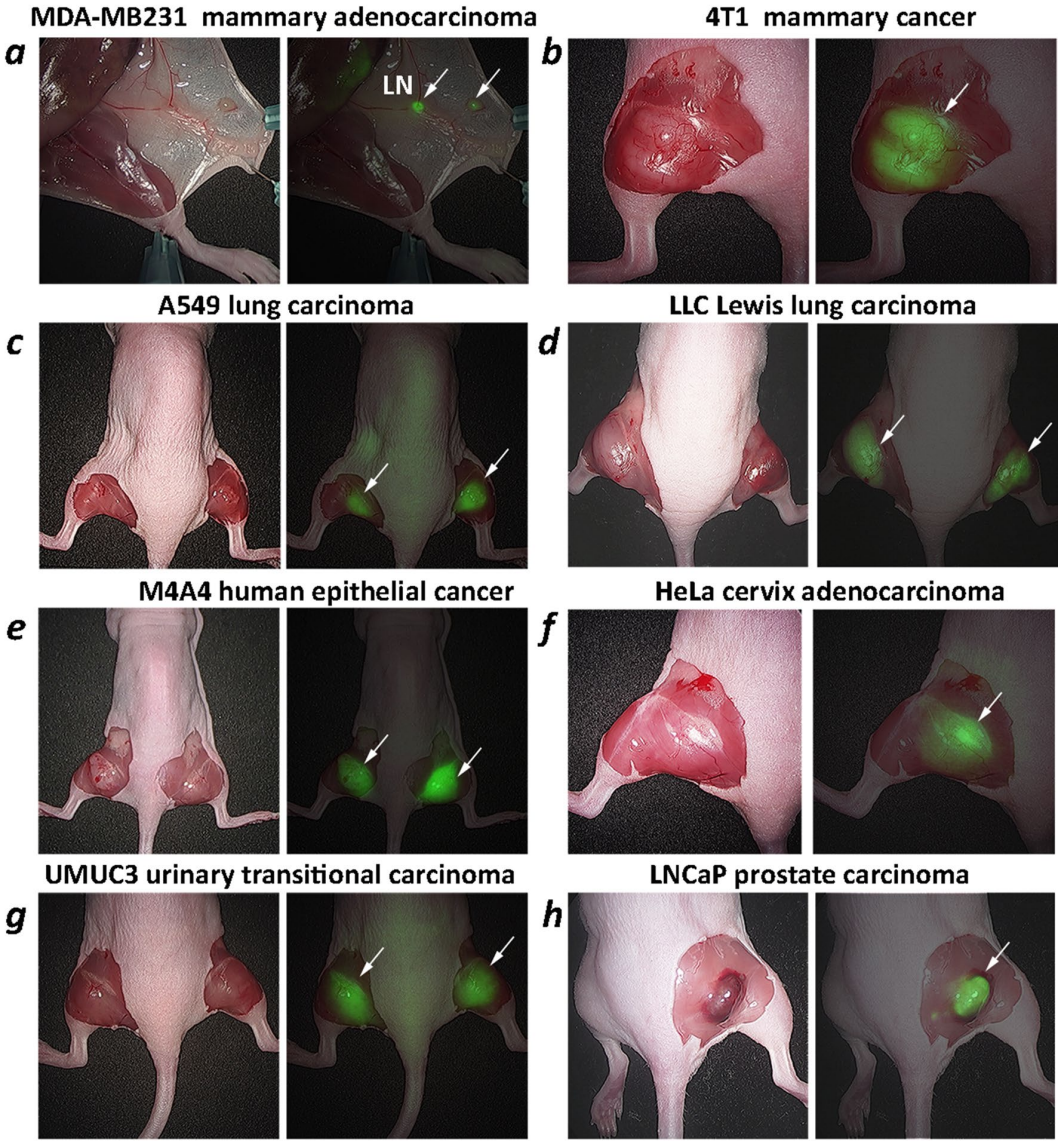
Tumor targeting. Representative photos and overlay of photos and in vivo NIR pHLIP ICG fluorescent images of athymic female nude mice bearing murine and human tumors are shown with skin removed from the tumor site. Targeting of human breast MDA-MB-231 tumor (**a**), murine breast 4T1 tumor (**b**), human A549 lung tumor (**c**), murine LLC Lewis lung carcinoma (**d**), human M4A4 epithelial tumor (**e**), human HeLa cervical tumor (**f**), human UMUC3 urinary bladder tumor (**g**) and human prostate LNCaP tumor (**h**) are shown. The imaging was performed at 24 h after a single i.v. administration of pHLIP ICG. Arrows indicate tumor locations. In the case of the MDA-MB231 breast tumor model, both a small tumor (arrow at the right) and an inguinal lymph node (indicated as LN) are targeted.

**Figure 6:**
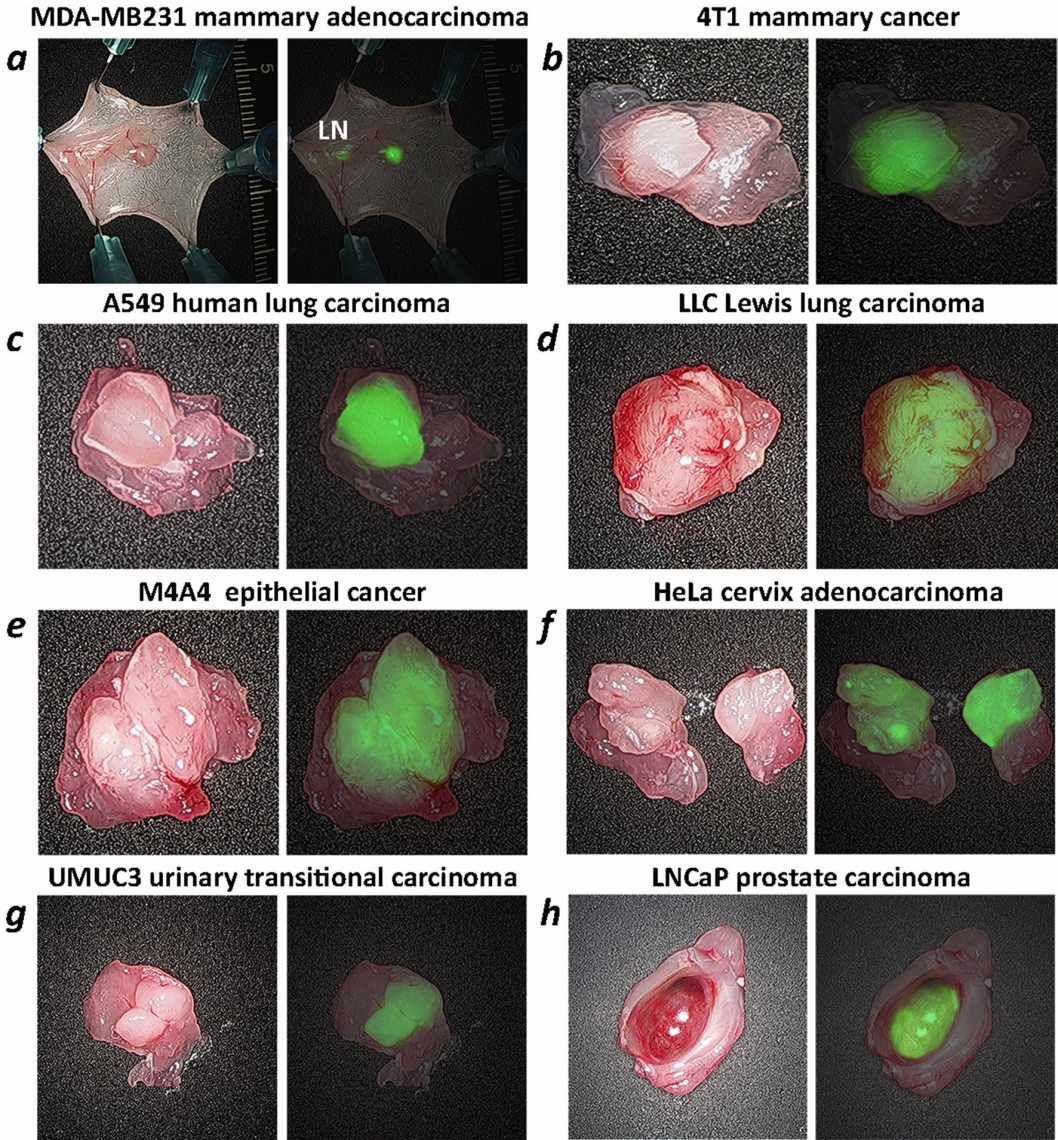
Ex vivo imaging of tumors with surrounding muscle. Representative photos, and overlays of photos and ex vivo NIR pHLIP ICG fluorescent images of tumors resected with surrounding tissue immediately after in vivo imaging. Targeting of human breast MDA-MB-231 tumor (**a**), murine breast 4T1 tumor (**b**), human A549 lung tumor (**c**), murine LLC Lewis lung carcinoma (**d**), human M4A4 epithelial tumor (**e**), human HeLa cervical tumor (**f**), human UMUC3 urinary bladder tumor (**g**) and human prostate LNCaP tumor (**h**) are shown. In the case of the MDA-MB231 breast tumor model, both a small tumor and an inguinal lymph node (indicated as LN) are targeted.

**Figure 7:**
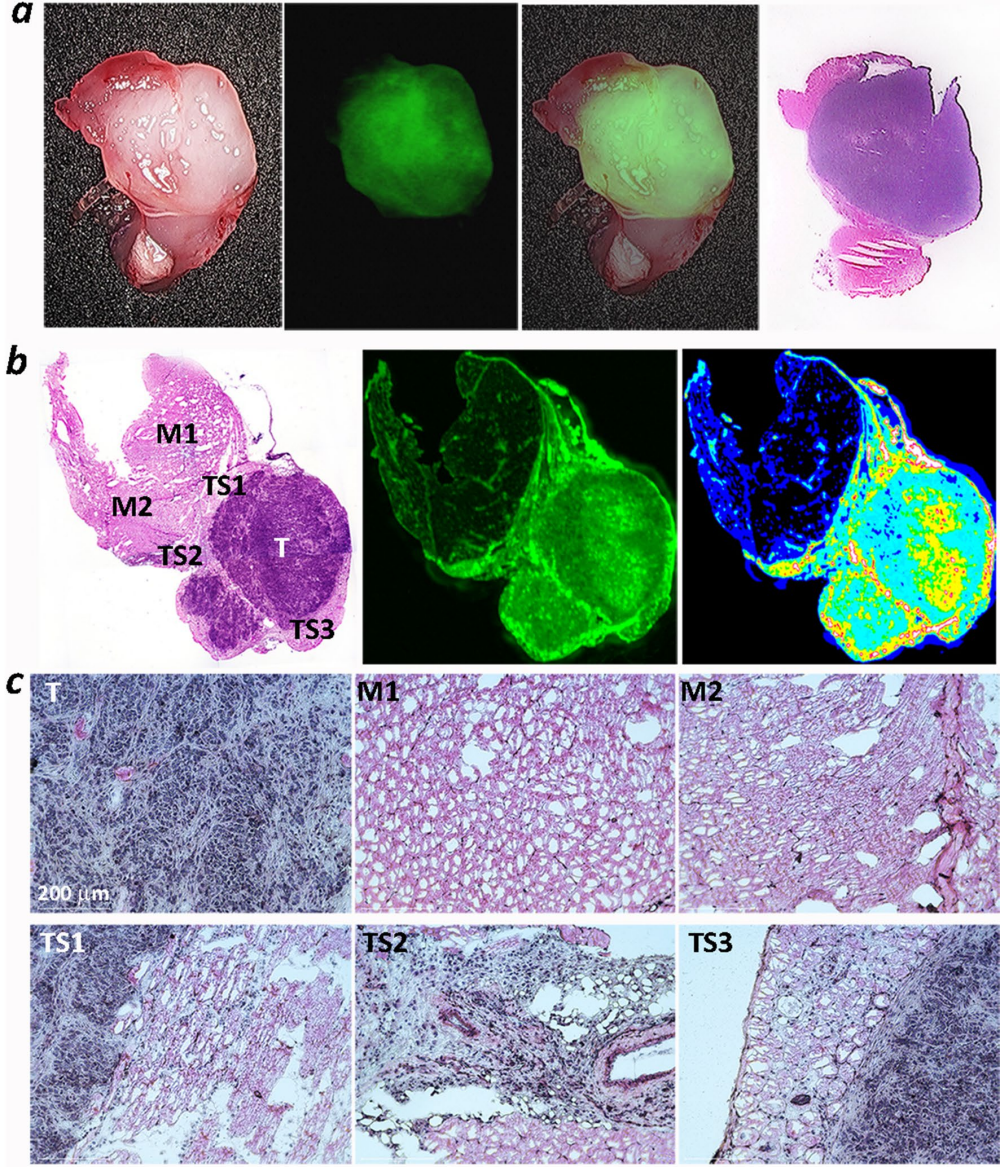
Ex vivo imaging and imaging of sections. (**a**) Representative images of tumors with surrounding tissue: photos, NIR pHLIP ICG fluorescent images, overlay of photos with NIR pHLIP ICG fluorescent images, and images of HE-stained sections obtained from the same tumor-muscle pieces are shown. (**b**) Bright field image of HE-stained section and NIR pHLIP ICG fluorescent images of the adjacent non-processed section obtained on IR scanner shown in monochrome (green) and 16-color (white and red are the highest intensity, and black and dark blue are the lowest intensity) presentations. T indicates tumor; M indicates muscle, and S indicates stroma. (**c**) Magnified bright field images (using 20 × objective) of tumor (T), muscle (M1 and M2), tumor stroma (TS1, TS2 and TS3) of HE-stained section are presented in panel (**b**).

pHLIP ICG tumor targeting was observed in several breast tumor models (MDA-MB-231 human and 4T1 murine, each is triple-negative), lung (A549 human and LLC murine), epithelial (melanoma) (M4A4 human), cervical (HeLa human), urinary bladder (UM-UC3 human) and prostate (LNCaP human) cancers. NIR fluorescence imaging on live animals using the Stryker clinical imaging system was performed at 24 h after a single i.v. administration of pHLIP ICG (Supplementary Figure SI,9). To closely mimic the conditions of intraoperative imaging, during which fluorescence is monitored from exposed tissue, the skin was removed from the tumor side while animals were under anesthesia. Excellent tumor targeting by pHLIP ICG was seen, as was a clear visualization of small, medium and large tumors positioned deep in the tissue or exposed at the surface, including small nearby micro-metastases (representative images are shown in Fig. 5). MDA-MB231 breast tumor cells were injected in the area of the mammary fat pads of athymic nude mice, and, most probably, drained into the inguinal lymph nodes (LN). Both the primary tumor site and the LN were targeted by pHLIP ICG (Figs. 5a, 6a). Previous studies show no targeting of inguinal LNs by a pHLIP peptide^25^, so the observed targeting is likely to be reporting cancerous LNs. Removal of the tumor and imaging of the tumor bed is shown in Supplementary Figure SI,10. To further examine the contrast between tumor and normal tissue, the tumors were removed with surrounding muscle tissue (Fig. 6). The border between tumor and muscle in most cases was evidently visible and was defined with high precision by the fluorescence signal. To correlate the pHLIP ICG NIRF signal with tumor location, the tumor-muscle pieces were frozen immediately following NIRF imaging, sectioned, fixed and stained with hematoxylin and eosin (HE). An excellent correlation between pHLIP ICG imaging and HE histopathology in locating the tumors is shown in Fig. 7. Fluorescent (unprocessed) and HE-stained sections were examined via an IR scanner and optical microscope. Tumors (marked as T) and surrounding tumor stroma (marked as TS1, TS2 and TS3) exhibit pHLIP ICG NIR fluorescent signal, as opposed to the muscles (marked as M1 and M2). Cancer cells infiltrating surrounding muscles are clearly seen in magnified images of tumor stroma, in agreement with previous findings^13^. The ability of pHLIP ICG to stain both tumor mass and tumor stroma is expected to improve margin resection during surgery.

### Fluorescence-guided resection of tumors

To establish the optimal concentration and timing after pHLIP ICG administration, surgical resection of the implanted tumors was performed at 24 and 48 h after pHLIP ICG tail vein injection at doses ranging from 0.5 to 2.5 mg/kg. The minimal dose of 0.5 mg/kg was sufficient for the successful visualization of tumors using a Stryker SPY-PHI handheld clinical instrument, which will be used in breast cancer surgical clinical trials for imaging of pHLIP ICG. The images and video obtained during the procedure are presented in Supplementary Figure SI,11 and Supplementary Video SI,3.

### GLP safety animal studies

The animal efficacy studies indicated that satisfactory imaging can be attained with a dose of 0.5 mg/kg of pHLIP ICG, which corresponds to a 0.04 mg/kg human dose (h.d.) per Body Surface Area (BSA)^26^. Various toxicology studies were performed on mice, dogs, rats and rabbits at doses of 25 × to 30 × the h.d., the maximum feasible dose (the details of these studies can be found in the Section “Pharmacology and Toxicology Studies” in the Supplementary Information). In summary, the following GLP studies were carried out with no adverse effects being observed, supporting the safety of translation of the agent to human trials:

#### Single-dose and 7-days repeat i.v. toxicity in beagle dogs

pHLIP ICG was administered by single i.v. bolus injection at increasing doses such as 0.063 mg/kg (0.88 × h.d. recalculated for dog dose), 1.08 mg/kg (15 × h.d.), 2.16 mg/kg (30 × h.d.) or once daily for 7 days at 2.16 mg/kg/day (30 × h.d. per day) to dogs, and did not adversely affect the overall health or condition of the animals, any of the measured clinical pathology parameters, organ weights, macroscopic or microscopic pathology. Based on these results, the no-observed-adverse-effect level (NOAEL) was above 2.16 mg/kg/day (30 × h.d. per day) for 7 days repeated administration of pHLIP ICG in males and females.

#### 7-day repeat i.v. toxicity in B6D2F1 mice

pHLIP ICG was administered once daily for 7 days at 4.92 mg/kg/day (10 × h.d. per day recalculated for mice dose), 7.38 mg/kg/day (15 × h.d. per day) and 12.3 mg/kg/day (25 × h.d. per day). The NOAEL was above 12.3 mg/kg/day (25 × h.d. per day) for 7 days repeated administration of pHLIP ICG in male and female mice.

#### A cardiovascular telemetry study in unrestrained, conscious non-naïve beagle dogs

A single administration of pHLIP ICG at dose levels of 0.072 mg/kg (1 × h.d. recalculated for dog dose per BSA), 0.36 mg/kg (5 × h.d.), and 1.44 mg/kg (20 × h.d.) to male dogs registered no effect on systemic blood pressures, heart rate, body temperature, electrocardiographic intervals, or qualitative ECG parameters up to 24 h post-dose.

#### Local tolerance/irritancy in New Zealand white rabbits

A single administration of pHLIP ICG at dose levels of 0.124 mg/kg (1 × h.d. recalculated for rabbit dose per BSA) and 1.86 mg/kg (15 × h.d.) through all routes of administration was well-tolerated and did not result in any pHLIP ICG-related changes.

#### A pharmacological safety assessment on the central nervous system in Sprague Dawley rats

A single administration of pHLIP ICG at dose levels of 0.218 mg/kg (0.88 × h.d. recalculated for rat dose per BSA), 0.248 mg/kg (1 × h.d.), 3.72 mg/kg (15 × h.d.) and 7.44 mg/kg (30 × h.d.) had no effect on the central nervous system up to 24 h post-dose.

#### Micronucleus test in Sprague Dawley rats

A single administration of pHLIP ICG at dose levels of 6.25 mg/kg (25 × h.d. recalculated for rat dose per BSA), 12.5 mg/kg (50 × h.d.) and 25 mg/kg (100 × h.d.) showed no evidence of genotoxic activity.

## Discussion

Fluorescence imaging, which has long been used to image blood flow, is finding a new range of applications in image-guided surgery. For decades, fluorescence angiography has been applied to assess blood flow and tissue perfusion in preoperative, intraoperative and postoperative settings^27–31^. Emerging uses in fluorescence-guided surgery promise to improve surgical outcomes by revealing tumor margins and, even more importantly, by marking flat lesions and micro-metastases adjacent to primary tumors, which are difficult to identify^32–35^.

Several FDA-approved fluorescent dyes and compounds are now in clinical use. The most widely used is the near-infrared emitting ICG, first approved for angiography and subsequently used in ophthalmology and elsewhere for imaging blood vessels^36–41^. ICG circulates in blood for a few minutes (2.5 min half-life)^42,43^ and is cleared through the liver, appearing in the bile about 8 min after injection^37^. If administered intraperitoneally, ICG is found in the nearest lymph nodes within 15 min, and in the regional lymph nodes within 1–2 h^44,45^. The IV dose of ICG varies from 0.5 to 2.0 mg/mL/kg of body weight. At a high dose of 5 mg/kg, no acute toxicity has been observed^37^ and chronic toxicity has not been reported over decades of many clinical uses of ICG.

Notwithstanding the utility that fluorescence-guidance using ICG has afforded to surgical procedures, there remain several important limitations associated with its use. ICG (as any non-targeted small molecule) does not strongly accumulate in tumors except for a very weak signal from enhanced permeation retention (EPR) in vascularized tumors. ICG may be taken up by some cancerous lesions only if blood flow uptake is enhanced for the particular lesion. In other cases, ICG has been used to visualize a lesion via a lack of signal, where the uptake/signal of ICG was much lower within the lesion compared to the surrounding tissue^46^. However, ICG does not have any inherent tumor-targeting properties based on selective binding. Also, the use of ICG for steady-state blood vessels visualization is limited by its very fast blood clearance profile, which can necessitate multiple injections in a single surgical procedure. pHLIP ICG resolves these deficiencies.

Tumor targeting by pHLIP peptides has been well-documented in a large variety of animal tumor models and human tissues. pHLIP ICG was used to image cancerous lesions in human bladders^19^ and the human upper urinary tract^47^. The agent was applied ex vivo after the surgical removal of organs, followed by washing and NIR fluorescent imaging using clinical imaging instruments. Malignant bladder lesions were targeted by pHLIP ICG and identified by NIR imaging; pathology showed a sensitivity of 97% and specificity of 100%. Carcinoma in situ was accurately diagnosed in 11 cases, whereas only four of these cases were seen using white light. All malignant upper tract lesions were targeted by pHLIP ICG and visualized on NIR imaging with a sensitivity of 100% (compared to 78.9% white light imaging) and a specificity of 100%. Benign collecting systems and ureters did not show uptake of the pHLIP construct.

Taken together, the published work on the mechanism of pHLIP peptides action, on tumor targeting and visualization, as well as in the preclinical studies with pHLIP ICG presented here, including pharmacology and toxicology assessments, motivate the clinical translation of pHLIP ICG for systemic administration for (i) real-time blood vessels visualization and (ii) targeting and identification of cancerous lesions during surgical procedures. Further, real-time blood vessels imaging with pHLIP ICG is expected to significantly extend the imaging time window from several minutes to several hours, allowing imaging to be performed at any time during a surgical procedure after a single injection of the agent. Potentially, multi-color imaging could be developed: pHLIP ICG could be used to visualize blood vessels, and pHLIP peptides with a fluorescent dye emitting in the range of 600–700 nm could be used for tumor imaging, allowing for the imaging of cancerous lesions and blood vessels during the same surgical procedure. It is expected that pHLIP ICG will allow accurate identification of tumor margins by staining of tumor mass and tumor stroma, targeting and visualization of flat lesions and, potentially, identification of micro-metastasis nearby primary tumor masses.

pHLIP ICG currently is in clinical translation for the fluorescence-guided surgical resection of breast tumors. According to the American Cancer Society, ~ 276,480 and ~ 48,530 new cases of invasive and non-invasive breast cancer will be diagnosed in US in 2020, and ~ 42,170 women will die from breast cancer. Surgery remains a dominant first approach to treatment, and there has been a significant shift in surgical practice toward breast-conserving surgery since 1980. The goal is to remove the tumor mass and, at the same time, preserve as much healthy tissue as possible. Unfortunately, ~ 25% of patients with invasive carcinoma and one-third of those with ductal carcinoma in situ require re-excision with about half of the re-excisions done in patients with negative margins, defined as “no ink on tumor”^48,49^. Two factors are known to have an important role: the process of identification of tumor negative margins and breast cancer biology. The tumor margin is first established intra-operatively (by eye), marked by ink, the tumor is resected, and, later, the “no ink on tumor margin” is confirmed by a pathologist. Difficulties in the marking of margins, specimen handling and pathology evaluation remain limitations to be overcome^50–52^. Pathology assessment takes time and the margin status is known only after surgery. Even when the margin is negative (as accurately as can be established), the local recurrence is still high, especially in the cases of triple-negative breast cancer, which test negative for estrogen receptor (ER), progesterone receptor (PR) and hormone epidermal growth factor receptor 2 (HER2), significantly limiting targeting and evaluations of this tumor type. In our study, we show targeting of triple-negative breast cancer in murine 4T1 and human MDA-MB-231 mouse models, including targeting of cancerous LNs. If our findings are successfully translated to the clinic, significant improvements of surgical outcomes should be expected.

## Supplementary information

Supplementary Information.

Supplementary Video 1.

Supplementary Video 2.

Supplementary Video 3.
