# Supplementary material for: pHLIP ICG for delineation of tumors and blood flow during fluorescence-guided surgery
Source: Sci Rep. 2020 Oct 27;10:18356. doi: 10.1038/s41598-020-75443-5 (PMC7591906; doi:10.1038/s41598-020-75443-5)
Supplement: Supplementary file 1 — Supplementary Information. [file 41598_2020_75443_MOESM1_ESM.docx]

**Supplementary Information**

**pHLIP ICG for delineation of tumors and blood flow during fluorescence-guided surgery**

Troy Crawford, Anna Moshnikova, Sean Roles, Dhammika Weerakkody, Michael DuPont, Lukas M. Carter, John Shen, Donald M. Engelman, Jason S. Lewis, Oleg A. Andreev, Yana K. Reshetnyak

## METHODS

## Manufacturing of pHLIP ICG

All manufacturing procedures were completed in accordance with good laboratory practice (GLP) quality control specifications. Synthesis of pHLIP ICG (NH_2_-Ala-Cys-Asp-Asp-Gln-Asn-Pro-Trp-Arg-Ala-Tyr-Leu-Asp-Leu-Leu-Phe-Pro-Thr-Asp-Thr-Leu-Leu-Leu-Asp-Leu-Leu-Trp-Ala-COOH) acetate salt (C_204_H_283_N_39_O_50_S_2_, M.W. 4,145.8 g/mol) was developed and the agent was produced by Iris Biotech, GmbH (Germany) in partnership with Chemical and Biopharmaceutical Laboratories (CBL) (Greece). The pHLIP peptide was synthesized on a solid support, followed by purification using HPLC. ICG-malemide was synthesized in solution, followed by column purification. ICG-malemide was conjugated with the pHLIP peptide in solution followed by purification of the final product (pHLIP ICG) using HPLC. Seven grams (7 g) of pHLIP ICG were produced with a purity of 98.7%, established by HPLC (Figures S1a); identity established by mass-spectrometry (m/z-ratio of the synthesized pHLIP ICG was 1383.0 Da [M+3H]^3+^/3 ± 1 and the expected theoretical mass is 1382.9 Da [M+3H]^3+^/3) was used in proof of concept (PoC), toxicology and pharmacokinetics studies. Manufacturing of pHLIP ICG and analytical characterization were further optimized by CordenPharma, GmbH (Germany). The HPLC analytical method developed for the detection of pHLIP ICG is the following: the HPLC column is from Waters, BEH C8, 150 x 2.1 mm, 1.7 µm particle size; the temperature is 33°C; the injection volume is 1.2 µL; eluent A is water + 0.04% TFA; eluent B is CH_3_CN + 0.04% TFA; flow rate is 0.4 mL/min; detection wavelength is 220 nm; and the following gradient:

| **Time, min** | **Eluent B, %** |
| --- | --- |
| 0 | 30 |
| 0.63 | 30 |
| 3.63 | 50 |
| 14.88 | 73 |
| 15.03 | 95 |
| 20.13 | 95 |
| 20.14 | 30 |
| 27.63 | 30 |

A spiking experiment was used to compare the pHLIP ICG product obtained after optimization of the manufacturing process with previously produced material. Both pHLIP ICG products were mixed in a ratio of 1:1 and analyzed by HPLC (Figure S1b). The presence of a single signal on the HPLC chromatogram confirms the identity of pHLIP ICG products. A m/z-determination of the material produced was performed by UHPLC-MS. The analysis gave the expected m/z-ratio of 1381.96 Da [M+3H]^3+^/3 (Figure S1c). GLP pHLIP ICG verification batch #1912127 was manufactured in Frankfurt by CordenPharma, the acceptance criteria are presented in Supplementary Table S1.

##

## Stability Study

Stability studies were performed with pHLIP ICG formulations in PBS and PBS containing 5% DMSO or 5% Ethanol (vol/vol). The tumor targeting capability in mice is independent of formulation. Formulation of pHLIP ICG in PBS/5% Ethanol was used in PoC animal studies, and in toxicity studies on mice, rats and dogs. For the PBS/5%DMSO formulation 1 mg of the lyophilized powder of pHLIP ICG was dissolved in 75 µl of DMSO (to make 3.2 mM solution), then 10 µl of 3.2 mM stock was mixed with 190 µl PBS to make a 0.16 mM solution of pHLIP ICG (5% DMSO). For the PBS/5%Ethanol formulation, the lyophilized powder of pHLIP ICG was dissolved at a concentration of 1.6 mg/ml in PBS/5% Ethanol solution. Finally, the formulation selected for human use was prepared by dissolving the lyophilized powder of pHLIP ICG at a concentration of 0.8 mg/ml in PBS. All formulations were kept at room temperature and protected from light. The aliquots were taken at 0.5 or 1, 3, 6, 24, 48 and 72 hours (102 hrs for pHLIP ICG dissolved in PBS) for analytical HPLC analysis using a Zorbax SB-C18 column (4.6 x 250 mm, 5 µm) with a binary solvent system using a 15­85% water and acetonitrile gradient with 0.05% TFA over 25 min. All formulations demonstrated stability (> 95% purity) up to 72 hours when kept at room temperature (RT). Formulation of pHLIP ICG in PBS was stable in solution for 4 days kept at RT.

**Liposome Preparation**

POPC (1-palmitoyl-2-oleoyl-sn-glycero-3-phosphocholine, Avanti Polar Lipids, Inc.) liposomes were used in biophysical measurements to mimic cell membranes and investigate the pHLIP ICG interactions with lipid bilayers. Large unilamellar vesicles (LUVs) were prepared by extrusion. POPC dissolved in chloroform was desolvated in a rotary evaporator and placed under high vacuum for 2 hours to create a phospholipid film. Lipids were then rehydrated in phosphate buffer (pH8) and repeatedly extruded through membranes with a pore size of 100 nm to make LUVs.

**Absorption and Emission Measurements**

Absorption spectra of pHLIP ICG, Methylene Blue (MB) and Isosulfan Blue (IB) in PBS, methanol, DMSO or DMF were measured in a cuvette with 1 cm path length using a Genesys 10S UV-Vis (Thermo Scientific) spectrophotometer. Fluorescence spectra of pHLIP ICG (with an excitation of 295 nm to record tryptophan fluorescence of pHLIP peptide, and 805 nm to record ICG fluorescence), MB (with an excitation of 670 nm) and IB (with an excitation of 630 nm, the fluorescence was insignificant) were measured in aqueous solution (in the presence or absence of POPC liposomes in the case of pHLIP ICG), methanol, DMSO or DMF using PC1 (ISS, Inc) and SpectraMax M2 (Molecular Devices) spectrofluorometers.

**Calculation of Molar Attenuation Coefficient**

Different amounts of pHLIP ICG (1.85, 1.808, 3.90, 4.15, 4.191, 7.16 and 7.19 mg) were weighed and dissolved in 2 ml of methanol followed by 25x or 50x further dilution in methanol for measurements of absorbance spectra (each spectra were recorded several times). The absorbance spectra were recorded from 700 to 900 nm. It was ensured that the absorbance was zero at 900 nm. All spectra were normalized and adjusted for the peptide content in the sample. The OD reading at 810 nm was used to calculate the molar attenuation coefficient according to the equation:

$$\varepsilon_{810}=\frac{{OD}_{810}}{c\cdot l}$$

where *c* is the concentration of pHLIP ICG calculated by weight and *l* is the optical path of the cuvette (1 cm). The mean molar attenuation coefficient for pHLIP ICG in methanol at 810 nm was established to be 152,533 ± 5,225 M^-1^ cm^-1^.

**Circular Dichroism and pH-Dependence**

Circular dichroism (CD) measurements were employed to monitor the pH-dependent insertion of pHLIP ICG into the membrane of POPC liposomes and the formation of helical structure. CD measurements were performed using a MOS-450 spectrometer (Biologic, Inc) with its temperature control set to 25.0°C. CD spectra were recorded from 200 to 260 nm with steps of 1 nm. The pH-dependent insertion of pHLIP ICG into the lipid bilayer of POPC liposomes was studied by monitoring the changes in the molar ellipticity (measured in millidegrees) at 222 nm as a function of pH. After the addition of aliquots of citric acid, the pHs of solutions containing 5 μM pHLIP ICG and 750 µM POPC liposomes were measured using an Orion PerHecT ROSS Combination pH Micro Electrode and an Orion Dual Star pH and ISE Benchtop Meter before and after each spectrum measurement to ensure that equilibrium is achieved. The ellipticity millidegrees were plotted as a function of pH. The pH-dependence was fit with the Henderson-Hasselbalch equation (using OriginLab software) to determine the cooperativity ($n$) and the mid-point ($pK$) of a transition:

$$Normalized pH dependence=\frac{1}{1+{10}^{n(pH-pK)}}$$

**Kinetics Measurements**

To follow pHLIP ICG insertion into the lipid bilayer of POPC liposomes in real-time, kinetics measurements were performed using a SFM-300 mixing system (Bio-Logic Science Instruments) in combination with a MOS-450 spectrometer with its temperature control set to 25.0°C. pHLIP ICG pre-mixed with POPC liposomes in phosphate buffer at pH8 were mixed (5 ms dead time) with acid to drop the pH from pH8 to pH5 and promote pHLIP peptide insertion into the bilayers. The insertion process was monitored by recording changes of the tryptophan fluorescence of pHLIP peptide excited at 295 nm and using a cut off filter at 320 nm.

## Cytotoxicity

Human mammary epithelial cells (HMEpC) acquired from Cell Applications, Inc. were authenticated and stored according to supplier’s instructions. Cells were cultured in mammary epithelial cell growth medium provided by the manufacturer. HMEpC cells were loaded in the wells of 96-well plates (~6,000 cells per well) and incubated overnight. Increasing amounts of pHLIP ICG dissolved in cell growth medium were added to cells to give the following final concentrations of pHLIP ICG with cells: 0.125, 0.25, 0.5, 1, 2, 4, 8 and 16 μM. After 48 and 72 hours of incubation, a colorimetric reagent (CellTiter 96 AQ_ueous_ One Solution Assay, Promega) was added for 2 hours followed by measuring absorbance at 490 nm to assess cell viability. All samples were prepared in triplicate and each experiment was repeated several times.

## Hemolysis assay

Single donor human whole blood was purchased from Innovative Research, Inc. Red blood cells (RBCs) were collected by centrifugation of whole blood at 2000 rpm for 10 minutes followed by washing three times with Dulbecco’s PBS (DPBS) and re-suspended in DPBS at a concentration of 7.5% (vol:vol). Varying concentrations of pHLIP ICG (0.075, 0.15, 0.3, 0.6, 1.2 nmol) in DPBS were added to RBCs to give a 5% RBC suspension (the total volume of the solution with RBC was 150 µL). The resultant mixtures were incubated at 37°C for 2 hours and then centrifuged at 1500 RCF (~3000 RMP) for 10 min. Hemolysis was assessed by the release of hemoglobin, which was monitored by measuring absorbance at 450 nm of the supernatant hemoglobin. DPBS was used as a negative control. As positive controls, which result in 100% lysis of RBCs, we used (i) water and (ii) 10% Triton X-100. The percentage of hemolysis was calculated as follows:

$$\% Hemolysis=100\cdot\frac{{OD}_{Test}-{OD}_{NC}}{{OD}_{PC}-{OD}_{NC}}$$

where, *OD_Test_*, *OD_NC_*, and *OD_PC_* are the optical density reading (absorbance) values of the test sample, negative control and positive control, respectively. The assay was performed in triplicate. The amount of RBC lysis was less than 2% in all samples. For reference, in a mouse study 2.5 nmol of pHLIP ICG is injected per mouse (a 20-25 g mouse has about 1.2 mL of blood), or 2.08 nmol/ml (the dose in humans is much lower), while in a hemolysis assay the maximum tested concentration was 8 nmol/ml.

## Enzyme Binding Assay

The pHLIP ICG enzyme binding assays were performed by Eurofins Panlabs, Inc. according to standard assay protocols. The following enzymes were used in the binding assay with 2 µM of pHLIP ICG : acetyl cholinesterase; peptidase (angiotensin converting enzyme); ATPase (Na^+^/K^+^, pig heart); CTSG peptidase (cathepsin G); cyclooxygenases COX-1 and COX-2; monoamine oxidases MAO-A and MAO-B; phosphodiesterases PDE3 and PDE4D2; protein tyrosine kinase (insulin receptor); protein tyrosine kinase (LCK); protein serine/threonine kinase (PKC, non-selective); adenosine A1 and A2A; adenosine transporter; adrenergic α1A, α1B, α1D, α2A, α2B, β1 and β2; norepinephrine transporter (NET); androgen (testosterone); angiotensin AT1; bradykinin B2; calcium channels L-type (benzothiazepine, dihydropyridine, phenylalkylamine); calcium channel N-type; cannabinoid CB1 and CB2; chemokine CCR1; cholecystokinin CCK1 (CCKA) and CCK2 (CCKB); dopamine D1, D2L and D2S; dopamine transporter (DAT); endothelin ETA; estrogen ERα; GABA transporter; GABAAs (flunitrazepam, central; Ro-15-1788, hippocampus and chloride channel, TBOB); glucocorticoid; glutamate (AMPA, kainate, agonism NMDA, glycine NMDA, phencyclidine NMDA, polyamine NMDA, and mGlu5 metabotropic); glycine (strychnine-sensitive); histamine H1 and H2; chemokine CXCR2 (IL-8RB); cysteinyl leukotriene (CysLT1); melanocortin MC1 and MC4; muscarinic M1, M2, M3, and M4; tachykinin NK1; neuropeptide Y Y1; nicotinic acetylcholine α1 (bungarotoxin) and α3β4; opiate δ1 (OP1, DOP), κ (OP2, KOP), and μ (OP3, MOP); platelet activating factor (PAF); potassium channels (KATP and hERG); PPARγ; serotonin (5-hydroxytryptamine) 5-HT1A, 5-HT1B, 5-HT2A, 5-HT2B, 5-HT2C, and 5-HT3; serotonin transporter (SERT); sodium channel (site 2); vasopressin V1A; progesterone (PR-B). The most significant inhibitory effect of pHLIP ICG was observed on progesterone B and was followed by an additional study to identify *IC_50_* values, which were determined by a non-linear least square regression analysis using MathIQTM (ID Business Solutions Ltd., UK). The *K_i_* values were calculated by the equation from Cheng and Prusoff [Cheng, Y., Prusoff, W.H., Biochem. Pharmacol. 22:3099-3108, 1973] using the observed *IC_50_* of pHLIP ICG , the concentration of radioligand employed in the assay, and the historical values for the *K_D_* of the ligand (Eurofins Panlabs data). The Hill coefficient (*n_H_*), defining the slope of the competitive binding curve, was calculated using MathIQTM. The *IC_50_,* *K_i_,* and *n_H_* values were established for pHLIP ICG and compared to the known progesterone agonist, R-2050 (Figure S3).

**Pharmacology and Toxicology Studies**

Pharmacology and toxicology studies were performed at the Memorial Sloan Kettering Cancer Center (MSK) Antitumor Assessment Core Facility and at Charles River Labs (CRL) under approved animal protocols and in compliance with the Department of Health and Human Services, Food and Drug Administration (FDA), United States Code of Federal Regulations, Title 21, Pad: 58: Good Laboratory Practice for Nonclinical Laboratory Studies and as accepted by Regulatory Authorities throughout the European Union (OECD Principles of Good Laboratory Practice), and other countries that are signatories to the OECD Mutual Acceptance of Data Agreement.. The minimum human dose (h.d.) of pHLIP ICG is 0.04 mg/kg. All doses of pHLIP ICG used in pharmacology and toxicology studies were calculated based on the minimum h.d.

***Pharmacokinetics of pHLIP ICG in Beagle dogs following a single i.v. bolus injection***

The objective of this study performed at CRL was to characterize the pharmacokinetic profile of pHLIP ICG in Beagle dogs following a single i.v. injection.

The study design was as follows:

| Group No. | Test Material | Targeted Dose Level (mg/kg) | Achieved Dose Level^a^ (mg/kg) | Dose Volume (mL/kg) | Achieved Dose Concentration (mg/mL) | No. of Males |
| --- | --- | --- | --- | --- | --- | --- |
| 1 | pHLIP ICG | 0.072 | 0.064 | 2.5 | 0.025 | 3 |

^a^ achieved dose levels calculated based on 87.7% mean recovery results from formulation analysis.

Blood samples were collected at 5, 15, and 30 minutes, and 1, 2, 4, 6, 8, 12, 24, and 48 hours post-dose for pharmacokinetic evaluation. Samples were placed on crushed wet ice until centrifugation, which was carried out immediately. The samples were centrifuged as per standard procedures. An aliquot of 250 µL of the resultant plasma was separated, transferred to 2.0 mL low-bind tubes containing 1.25 mL of methanol, mixed and frozen immediately over dry ice and transferred to a freezer at -80°C. At the time of analysis, samples were thawed, extracted, and analyzed via LC-MS/MS according to the validated protocol using pHLIP_IS_.

**In conclusion**, the administration of a single dose of pHLIP ICG by i.v. bolus injection was well-tolerated in dogs at a dose level of 0.064 mg/kg. Evidence of systemic exposure to pHLIP ICG was observed in all animals and was quantifiable up to 8 or 12 hours post-dose. The mean C_0_ (theoretical concentration at time zero following a bolus dose administration), AUC_0‑48hr_ (area under the concentration-time curve from time zero to 48hrs) and AUC_INF_ (area under the concentration-time curve from time zero extrapolated to infinity) values were 1410 ng/mL, 5450 hr*ng/mL, and 5550 hr*ng/mL, respectively.

***Pharmacokinetic testing of pHLIP ICG in mice***

The purpose of this study, performed at MSK, was to determine the pharmacokinetic parameters of pHLIP ICG in 3 female B6D2F1 mice following a single i.v. injection of pHLIP ICG at a dose of 12.3 mg/kg. Blood was collected in EDTA tubes at 5, 15, 30 minutes, and 1, 2, 4, 6, 8, 16, and 24 hours post-injection, followed by centrifugation to separate the plasma, and flash frozen. At time of analysis, samples were thawed, extracted, and analyzed via LC-MS as per the validated protocol using pHLIP_IS_.

**In conclusion**, administration of a single dose of pHLIP ICG by i.v. bolus injection was well tolerated in mice at a dose level of 12.3 mg/kg. Evidence of systemic exposure to pHLIP ICG was observed in all animals and was quantifiable up to 16 hours post-dose.

***Single i.v. toxicity with acute (24 hrs) and delayed (14 days) necropsy and TK in Beagle dogs; 7-days repeat i.v. toxicity in dog with acute (24h after last injection) and TK on days 1 and 7 in Beagle dogs***

The objectives of this GLP study, performed at CRL, were to determine the potential toxicity of pHLIP ICG, when given by single dose i.v. injection or once daily (i.v. injections) for 7 days in dogs, and to evaluate the potential reversibility of any findings following the single dose injection. In addition, toxicokinetic characteristics of pHLIP ICG were examined.

The study design was as follows (40 Beagle dogs: 20 male and 20 female):

| Group No. | Test Material | Dose Level (mg/kg/day) | Dose Volume (mL/kg) | Dose Concentration (mg/mL) | No. of Animals | | | |
| --- | --- | --- | --- | --- | --- | --- | --- | --- |
|  |  |  |  |  | Main Study | | Recovery Study | |
|  |  |  |  |  | Males | Females | Males | Females |
| 1 | Vehicle^*^ | 0 | 2.5 | 0 | 3 | 3 | 2 | 2 |
| 2 | pHLIP ICG | 0.072^**^ | 2.5 | 0.029 | 3 | 3 | - | - |
| 3 | pHLIP ICG | 1.08^***^ | 2.5 | 0.432 | 3 | 3 | - | - |
| 4 | pHLIP ICG | 2.16^****^ | 2.5 | 0.864 | 3 | 3 | 2 | 2 |
| 5 | Vehicle | 0 | 2.5 | 0 | 2 | 2 | - | - |
| 6 | pHLIP ICG | 2.16 | 2.5 | 0.864 | 2 | 2 | - | - |

^*^ 5% (vol/vol) EtOH in PBS, pH 7.4

^**^ based on dose formulation analytical results, animals from Group 2 received a dose level of 0.063 mg/kg, which is equivalent to 0.875x h.d.

^***^ equivalent to 15x h.d.

^****^ equivalent to 30x h.d.

The following parameters and end points were evaluated in the study: mortality, clinical observations, body weights, food consumption, ophthalmology and electrocardiology assessments, clinical pathology parameters (hematology, coagulation, clinical chemistry, and urinalysis), toxicokinetic parameters, gross necropsy findings, organ weights, and histopathological examinations.

There were no unscheduled deaths. There were no pHLIP ICG -related clinical observations, and no pHLIP ICG -related effects on the assessment of body weights, food consumption, ophthalmology and electrocardiology changes, hematology, coagulation, and urinalysis parameters, organ weights, or macroscopic and microscopic evaluations at any dose level tested following a single dose or a daily 7-day repeat dose administration.

Administration of pHLIP ICG to dogs when given by repeated i.v. injection at 2.16 mg/kg/day for 7 days resulted in non-adverse clinical chemistry changes limited to minimal decreases in cholesterol in both males and females.

Systemic exposure to pHLIP ICG appeared to be independent of sex. Following a single i.v. bolus administration of pHLIP ICG, mean C_0_ and AUCT_last_ values increased with increasing dose in an approximately dose proportional manner from 1.08 to 2.16 mg/kg. Following daily i.v. bolus administration of 2.16 mg/kg pHLIP ICG, mean C_0_ and AUC_Tlast_ values were 38,400 ng/mL and 65,400 hr*ng/mL, respectively, on day 1 and were 42,700 ng/mL and 72,300 hr*ng/mL, respectively, on day 7. Systemic exposure (AUC_Tlast_) to pHLIP ICG did not appear to increase following repeated IV bolus administration of 2.16 mg/kg pHLIP ICG.

**In conclusion**, pHLIP ICG administered by a single i.v. bolus injection at 0.063, 1.08, or 2.16 mg/kg or once daily for 7 days at 2.16 mg/kg/day to dogs did not adversely affect the overall health or condition of the animals, and had no impact on clinical pathology parameters, organ weights, macroscopic or microscopic pathology. Decreases in cholesterol levels were noted in pHLIP ICG treated animals at 2.16 mg/kg/day after repeated dosing but were considered non‑adverse. Based on these results, the no-observed-adverse-effect level (NOAEL) was established and should be considered to be above of 2.16 mg/kg/day for 7 days repeated administration of pHLIP ICG, with a mean C_max_ of 40,300 ng/ml and AUC_0-24_ of 322,000 hr*ng/ml in males and females.

***7-day repeat i.v. toxicity in mice with acute (24 hrs after last injection) and delayed (14 days) necropsy and TK on days 1 and 7 in B6D2F1 mice***

The purpose of this GLP study performed at MSK was to assess the acute and delayed toxicity of a repeat administration of pHLIP ICG in mice. The pHLIP ICG was administered for 7 consecutive days.

The study design was as follows (82 B6D2F1 mice: 41 male and 41 female):

| Group No. | Test Material | Dose Level (mg/kg/day) | No. mice/group | Sex | Sacrifice | Necropsy |
| --- | --- | --- | --- | --- | --- | --- |
| Interim sacrifice on Days 8 and 9 | | | | | | |
| 1 | Vehicle^*^ | 0 | 10 | Male | Day 8 | Yes |
| 2 | pHLIP ICG | 13.6^**^ | 10 | Female |  |  |
| 3 | Vehicle | 0 | 10 | Male | Day 9 |  |
| 4 | pHLIP ICG | 13.6 | 10 | Female |  |  |
| Final sacrifice on Days 21 and 22 | | | | | | |
| 5 | Vehicle | 0 | 5 | Male | Day 21 | Yes |
| 6 | pHLIP ICG | 13.6 | 5 | Female |  |  |
| 7 | Vehicle | 0 | 5 | Male | Day 22 |  |
| 8 | pHLIP ICG | 13.6 | 5 | Female |  |  |
| TK samples | | | | | | |
| 9 | Vehicle | 0 | 3 | Male | Day 7 | No |
| 10 | pHLIP ICG | 13.6 | 3 | Female |  |  |
| 11 | Vehicle | 0 | 3 | Male | Day 8 |  |
| 12 | pHLIP ICG | 13.6 | 3 | Female |  |  |

^*^ 5% (vol/vol) EtOH in PBS, pH 7.4

^**^ equivalent to 27.6x h.d.

Throughout the study, body weights, clinical signs, clinical pathology (hematology and clinical chemistry), and histopathology were collected.

**In conclusion**, the repeat administration of pHLIP ICG may be associated with germ cell degeneration in males with incidence and severity decreasing over time. No other signs of systemic or delayed toxicity were observed.

***Follow up 7-days repeat i.v. toxicity in male mice with acute (24 hrs after last injection) and delayed (14 days) necropsy and TK on days 1 and 7 in B6D2F1 mice***

The purpose of this GLP study performed at MSK was to assess the acute and delayed toxicity on the male reproductive system of mice of a repeat administration of pHLIP ICG at different concentrations. The pHLIP ICG was administered once/day for 7 consecutive days.

The study design was as follows (77 male B6D2F1 mice):

| Group No. | Test Material | Dose Level (mg/kg/day) | No. of Male mice/group | Sacrifice | Necropsy |
| --- | --- | --- | --- | --- | --- |
| Interim sacrifice on Day 8 | | | | | |
| 1 | Vehicle^*^ | 0 | 10 | Day 8 | Yes |
| 2 | pHLIP ICG | 4.92^**^ | 10 |  |  |
| 3 | pHLIP ICG | 7.38^***^ | 10 |  |  |
| 4 | pHLIP ICG | 12.3^****^ | 10 |  |  |
| Final sacrifice on Day 21 | | | | | |
| 5 | Vehicle | 0 | 5 | Day 21 | Yes |
| 6 | pHLIP ICG | 4.92 | 5 |  |  |
| 7 | pHLIP ICG | 7.38 | 5 |  |  |
| 8 | pHLIP ICG | 12.3 | 5 |  |  |
| TK samples | | | | | |
| 9 | Vehicle | 0 | 3 | Day 7 | No |
| 10 | pHLIP ICG | 4.92 | 3 |  |  |
| 11 | pHLIP ICG | 7.38 | 3 |  |  |
| 12 | pHLIP ICG | 12.3 | 3 |  |  |

^*^ 5% (vol/vol) EtOH in PBS, pH 7.4

^**^ equivalent to 10x h.d.

^***^ equivalent to 15x h.d.

^****^ equivalent to 25x h.d.

Throughout the study, body weights, clinical signs, and histopathology were collected.

**In conclusion**, the repeat administration of pHLIP ICG at all dose levels is well tolerated, with no indication of germ cell degeneration or other toxicity to the male reproductive system.

***Local tolerance/irritancy in New Zealand white*** ***rabbits***

The objective of this GLP study performed at CRL was to compare the i.v., intra-arterial, perivascular and subcutaneous routes for potential irritation following a single injection of pHLIP ICG in the rabbits.

The study design was as follows (18 male New Zealand white rabbits):

| Group No. | Test Material | Targeted Dose Level (mg/kg) | Dose per 3kg Rabbit (mg) | Dose Volume (mL/kg) | | Dose Conc. (mg/mL) | Total injected Dose Based on 3kg Rabbit (mg) | No. of Males |
| --- | --- | --- | --- | --- | --- | --- | --- | --- |
|  |  |  |  | i.v. right ear vein | |  |  |  |
| 1 | Vehicle^*^ | 0 | 0 | 3 | | 0 | 0 | 3 |
| 2 | pHLIP ICG | 0.124^**^ | 0.372 | 3 | | 0.041 | 0.372 | 3 |
| 3 | pHLIP ICG | 1.86^***^ | 5.58 | 3 | | 0.62 | 5.58 | 3 |
|  | | | | i.a. left ear artery/  Subc.lumbar | Subc. left perivenous space |  | | |
| 4 | Vehicle | 0 | 0 | 1.5/1.5 | 0.25 | 0 | 0 | 3 |
| 5 | pHLIP ICG | 0.124 | 0.372 | 1.5/1.5 | 0.25 | 0.041 | 0.379 | 3 |
| 6 | pHLIP ICG | 1.86 | 5.58 | 1.5/1.5 | 0.25 | 0.62 | 5.735 | 3 |

i.a. = intra-arterial; i.v. = intravenous; subc. = subcutaneous.

^*^ 5% (vol/vol) EtOH in PBS, pH 7.4

^**^ equivalent to 1x h.d.; based on dose formulation analytical results, animals from Group 2 and 5 received a dose level of 0.109 mg/kg for i.v.,i.a. and/or subc. injection and 0.009 mg for perivenous space injection.

^***^ equivalent to 15x h.d.

The following parameters and end points were evaluated in this study: clinical signs, local irritation assessment, body weights (for dose calculation only), and histopathology of the injection sites. There were no clinical signs, local irritation or any microscopic changes associated with pHLIP ICG during the study.

**In conclusion**, a single administration of pHLIP ICG via i.v., intra-arterial, perivenous and subcutaneous injection was well tolerated in rabbits at total dose levels of 0.124 and 1.86 mg/kg. Administration did not result in any pHLIP ICG related changes.

***A pharmacological safety assessment on the central nervous system of Sprague Dawley rats***

The objective of this GLP study performed at CRL was to evaluate the pharmacological effects of pHLIP ICG on the central nervous system following a single i.v. bolus injection in rats.

The study design was as follows (32 male Sprague Dawley rats):

| Group No. | Test Material | Dose Level (mg/kg) | Dose Volume (mL/kg) | Dose Concentration (mg/mL) | No. of Males |
| --- | --- | --- | --- | --- | --- |
| 1 | Vehicle^*^ | 0 | 5 | 0 | 8 |
| 2a | pHLIP ICG | 0.218^**^ | 5 | 0.0436 | 4 |
| 2b | pHLIP ICG | 0.248^***^ | 5 | 0.0496 | 4 |
| 3 | pHLIP ICG | 3.72^****^ | 5 | 0.744 | 8 |
| 4 | pHLIP ICG | 7.44^*****^ | 5 | 1.488 | 8 |

^*^ 5% (vol/vol) EtOH in PBS, pH 7.4

^**^ equivalent to 0.88x h.d.

^***^ equivalent to 1x h.d.

^****^ equivalent to 15x h.d.

^*****^ equivalent to 30x h.d.

Functional observation battery (FOB) tests were performed once prior to dosing and again at 5 minutes, 3 and 24 hours post-dose and included the following assessments: palpebral closure, eye prominence, pupil size, pupillary response, lacrimation, salivation, body tone, extensor thrust, pinna reflex, tactile reflex, overall animal reactivity, auricular startle, air righting reflex, body temperature (rectal). Additional evaluations consisted of mortality and clinical observations.

There were no pHLIP ICG related changes on any qualitative or quantitative FOB parameters at 0.218, 0.248, 3.72 and 7.44 mg/kg up to 24 hours post-dose.

**In conclusion**, a single i.v. bolus administration of pHLIP ICG at dose levels of 0.218, 0.248, 3.72 and 7.44 mg/kg to rats had no observed effect on the central nervous system up to 24 hours post-dose.

***A cardiovascular telemetry study in unrestrained conscious non-naïve Beagle dogs***

The objective of this GLP study performed at CRL was to evaluate the potential cardiovascular effects of pHLIP ICG in instrumented dogs when administered by i.v. bolus injection using a Latin square design.

Each of the four male dogs received a dose of Reference Item (5 % Ethanol in Phosphate buffered saline (PBS), pH of 7.4 ± 0.2) and three dose levels of pHLIP ICG (0.072, 0.36, and 1.44 mg/kg), with a 7-day washout period between each dose. The dose was administered by i.v. bolus injection at a dose volume of 2.5 mL/kg.

The study design was as follows (16 male Beagle dogs):

| Dosing Schedule (mg/kg) | | | | | | |
| --- | --- | --- | --- | --- | --- | --- |
| Animal No. | Dose 1 | | Dose 2 | Dose 3 | Dose 4 | |
| 1 | 0 | | 0.072 | 1.44 | 0.36 | |
| 2 | 0.072^*^ | | 0.36 | 0 | 1.44 | |
| 3 | 0.36^**^ | | 1.44 | 0.072 | 0 | |
| 4 | 1.44^***^ | | 0 | 0.36 | 0.072 | |
|  | | | | | | |
| Dose Level (mg/kg) | | Concentration (mg/mL) | | Dose Volume (mL/kg) | | No. of Males |
| 0 | | 0 | | 2.5 | | 4 |
| 0.072 | | 0.029 | | 2.5 | | 4 |
| 0.36 | | 0.144 | | 2.5 | | 4 |
| 1.44 | | 0.576 | | 2.5 | | 4 |

^*^ equivalent to 1x h.d.

^**^ equivalent to 5x h.d.

^***^ equivalent to 20x h.d.

The following cardiovascular parameters were evaluated for all animals from 2 hours prior and up to 24 hours post each dose: systemic blood pressures (systolic, diastolic, mean arterial, and pulse pressure), heart rate, body temperature, and electrocardiographic duration/intervals (PR, QRS, QT, and QTc). Qualitative evaluation of the electrocardiographic waveforms was performed twice prior to each dose (at least 30 minutes apart) and at 5, 15, and 30 minutes, 1, 2, 3, 4, 6, 12, and 24 hours post-dose. Additional evaluations consisted of mortality and clinical observations. There were no mortalities or pHLIP ICG related clinical signs throughout the course of the study.

**In conclusion**, a single i.v. bolus injection of pHLIP ICG at 0.072, 0.36, and 1.44 mg/kg to male dogs resulted in no observable effect on systemic blood pressures, heart rate, body temperature, electrocardiographic intervals (PR, QRS, QT or heart rate corrected QT [QTc]), or qualitative ECG parameters up to 24 hours post-dose.

***Micronucleus test in Sprague Dawley rats***

The objective of this GLP study performed at CRL was to determine the potential genotoxicity of pHLIP ICG when given by i.v. bolus injection to rats using the peripheral blood micronucleus test.

In order to determine the maximum tolerated dose, a dose-range finding test was performed prior to the main test, where male and female rats (3 animals/sex) were administered a single dose of pHLIP ICG by i.v. injection. The doses evaluated were 14 mg/kg and 25 mg/kg using 5% EtOH in PBS, pH 7.4 as the vehicle. Animals were observed for signs of toxicity and/or mortality. As no adverse clinical signs were noted during the dose‑range finding test, the maximum practical dose of 25 mg/kg was set as the high dose to be evaluated in the main test using a single sex (males).

The main phase study design was as follows (37 rats: 31 males and 6 females):

| Group No. | | Test Material | Dose Level (mg/kg) | Dose Volume (mL/kg) | Dose Concentration (mg/mL) | No. of Animals | |
| --- | --- | --- | --- | --- | --- | --- | --- |
|  |  |  |  |  |  | Male | Female |
| 1 | pHLIP ICG | | 14^***^ | 10 | 1.4 | 3 | 3 |
| 2 | pHLIP ICG | | 25^****^ | 10 | 2.5 | 3 | 3 |
| 3 | | NP^*****^ | - | 10 | 0 | 5  5  5 | - |
| 4 | | pHLIP ICG | 6.25^*^ | 10 | 0.625 | 5 | - |
| 5 | | pHLIP ICG | 12.5^**^ | 10 | 1.25 | 5 | - |
| 6 | | pHLIP ICG | 25 | 10 | 2.5 | 5 | - |
| 7 | CP^******^ | | 10 | 10 | 1 | 5 | - |

^*^ equivalent to 25x h.d.

^**^ equivalent to 50x h.d.

^***^ equivalent to 56x h.d.

^****^ equivalent to 100x h.d. – the maximum practical dose of PHLIP ICG

^*****^ NC is the negative control, 5% EtOH in PBS, pH 7.4.

^******^ PC is the positive control, cyclophosphamide monohydrate. Animals were dosed twice with CP by oral gavage with an interval of 24 hours (± 15 minutes).

Analysis of pHLIP ICG dosing formulations conducted during the study confirmed that all formulations are acceptable for use.

No mortalities occurred during the main test and no pHLIP ICG related adverse clinical signs were observed. Additionally, no pHLIP ICG related body weight loss or depression in body weight gains were observed.

Blood was collected for micronuclei evaluation at 45 hours post-dose (Groups 3 to 7), and 69 hours post-dose (Groups 3 and 7). The proportion of immature erythrocytes among total erythrocytes in the peripheral blood (%RETs) and the percentage of micronucleated reticulocytes (%MN-RETs) in the NC group were within the laboratory historical negative control range. A clear, unequivocal and statistically significant increase in micronuclei was observed in the PC group. The results from both NC and PC groups confirmed the validity of the assay.

No substantial reduction (*i.e.* less than 50% the value of the concurrent NC) in the %RETs was observed for any of the pHLIP ICG dosed groups, indicating that no substantial cytotoxicity was observed. Animals dosed with pHLIP ICG did not show any statistically significant increases in the %MN-RETs when compared to the concurrent NC group. Therefore, pHLIP ICG did not induce cytogenetic damage in peripheral blood immature erythrocytes in this micronucleus test, when administered once, by i.v. injection to male rats, up to the maximum practical dose of 25 mg/kg.

**In conclusion**, pHLIP ICG showed no evidence of genotoxic activity in this *in vivo* study, when tested in accordance with regulatory guidelines.

**In vitro *bacterial reverse mutation test***

The objective of this study performed at CRL was to determine the potential genotoxicity of pHLIP ICG using the bacterial reverse mutation test.

The experimental design was as follows:

| Dose No. | Formulation Conc.  (µg/mL) | Dose Volume (µL) | Final Conc. (µg/plate) | No. of Replicates | | No. of  Strains |
| --- | --- | --- | --- | --- | --- | --- |
|  |  |  |  | 0S9 | +S9 |  |
| Negative Control^*^ | - | 100 | - | 3 | 3 | 5 |
| 1/ pHLIP ICG | 500 | 3.16 | 1.58 | 3 | 3 | 5 |
| 1/ pHLIP ICG | 500 | 10 | 5.0 | 3 | 3 | 5 |
| 1/ pHLIP ICG | 500 | 31.6 | 15.8 | 3 | 3 | 5 |
| 1/ pHLIP ICG | 500 | 100 | 50 | 3 | 3 | 5 |
| 2/ pHLIP ICG | 1581 | 100 | 158 | 3 | 3 | 5 |
| 3/ pHLIP ICG | 5000 | 100 | 500 | 3 | 3 | 5 |
| 4/ pHLIP ICG | 15811 | 100 | 1581 | 3 | 3 | 5 |
| 5/ pHLIP ICG | 50000 | 100 | 5000^**^ | 3 | 3 | 5 |
| Positive controls | ^***^ | 100 | ^***^ | 3 | 3 | 5 |

^*^ vehicle, dimethyl sulfoxide (DMSO), was used as a negative control

^**^ the OECD/ICH S2(R1) standard limit dose (usually 5000 µg/plate).

^***^ dose depended on the test strain, the positive controls and methodology used.

*Salmonella typhimurium* strains (TA1535, TA1537, TA98, TA100 and TA102) were treated with pHLIP ICG at a range of concentrations up to 5000 µg/plate (the standard limit dose for this assay), in the presence and absence of a supplemented rat liver fraction (S9 mix), using the plate incorporation version of the bacterial reverse mutation test.

Bacteria were incubated with standard positive controls, and the response of the various bacterial strains to these agents confirmed the sensitivity of the test system and the activity of the S9 mix.

Incomplete, or absent, background lawns of non‑revertant bacteria, or substantial reductions in revertant colony counts, were not obtained following exposure to pHLIP ICG, indicating that pHLIP ICG was non‑toxic to the bacteria at the levels tested. No precipitation was observed in the assay.

No substantial increases in revertant colony numbers were obtained with any of the tester strains, following exposure to pHLIP ICG at any dose level, in either the presence or absence of S9 mix. Therefore, pHLIP ICG was considered to be negative for the induction of mutagenicity in this *in vitro* assay.

**In conclusion**, pHLIP ICG did not show any evidence of genotoxic activity in this *in vitro* mutagenicity assay when tested in accordance with regulatory guidelines.

**In vitro *micronucleus test***

The objective of this study performed at CRL was to determine the potential genotoxicity of pHLIP ICG using an *in vitro* mammalian cell micronucleus test in human peripheral blood lymphocytes.

The experimental design was as follows:

| Dose No. | Formulation Conc. (µg/mL) | Dose Volume (µL/culture) | Final Conc.  (µg/mL) | No. of Cultures | | |
| --- | --- | --- | --- | --- | --- | --- |
|  |  |  |  | 4 Hours (0S9) | 4 Hours (+S9) | 24 Hours (0S9) |
| Negative Control^*^ | - | 50 | - | 2 | 2 | 2 |
| 1/ pHLIP ICG | 500 | 10 | 1.00 | 2 | 2 | 2 |
| 1/ pHLIP ICG | 500 | 20 | 2.00 | 2 | 2 | 2 |
| 1/ pHLIP ICG | 500 | 40 | 4.00 | 2 | 2 | 2 |
| 2/ pHLIP ICG | 800 | 50 | 8.00 | 2 | 2 | 2 |
| 3/ pHLIP ICG | 1600 | 50 | 16.0 | 2 | 2 | 2 |
| 4/ pHLIP ICG | 3200 | 50 | 32.0 | 2 | 2 | 2 |
| 5/ pHLIP ICG | 6400 | 50 | 64.0 | 2 | 2 | 2 |
| 6/ pHLIP ICG | 12800 | 50 | 128 | 2 | 2 | 2 |
| 7/ pHLIP ICG | 25600 | 50 | 256 | 2 | 2 | 2 |
| 8/ pHLIP ICG | 50000 | 50 | 500** | 2 | 2 | 2 |
| Colcemid | 10 | 80 | 0.16 | 2 | - | - |
|  | 10 | 90 | 0.18 | 2 | - | - |
|  | 10 | 100 | 0.20 | 2 | - | - |
| Nocodazole | 20 | 50 | 0.20 | 2 | - | - |
|  | 25 | 50 | 0.25 | 2 | - | - |
|  | 30 | 50 | 0.30 | 2 | - | - |
| Cyclophosphamide | 1000 | 50 | 10 | - | 2 | - |
|  | 1500 | 50 | 15 | - | 2 | - |
| Mitomycin C | 10 | 50 | 0.10 | - | - | 2 |
|  | 20 | 50 | 0.20 | - | - | 2 |

^*^ vehicle, dimethyl sulfoxide (DMSO), was used as a negative control

^**^  where the high level = 0.5 mg/mL.

Human peripheral blood lymphocytes were treated with pHLIP ICG at levels up to the standard limit of 0.5 mg/mL. The high dose for micronucleus assessment was 0.5 mg/mL, as no pHLIP ICG related cytotoxicity or precipitation was observed in the assay. The negative control results were within the laboratory negative historical control range. Lymphocytes were also incubated with standard positive controls, which caused statistically significant increases in the proportion of cells with micronuclei, confirming the sensitivity of the test system and the activity of the S9 mix. All criteria for a valid assay were therefore met.

Cultures treated with pHLIP ICG did not show any statistically significant increases in the incidence of micronucleated binucleate cells. All results were within the distribution of the laboratory historical negative control data. Therefore, pHLIP ICG was considered to be negative for the induction of micronuclei in this *in vitro* assay.

**In conclusion**, pHLIP ICG did not show any evidence of genotoxic activity in the *in vitro* micronucleus test in human peripheral blood lymphocytes when tested in accordance with regulatory guidelines.

## Animal Imaging Studies

All imaging studies on mice were conducted at University of Rhode Island according to the approved animal protocol AN04-12-011 in compliance with the principles and procedures outlined by the National Institutes of Health for the care and use of animals. All imaging studies in pigs were conducted at the Porcine Laboratory, Sutter Institute for Medical Research and approved animal protocol STE.10.19 (Stryker Endoscopy Imaging and Instrumentation Studies).

## Imaging of Blood Vessels in Mice

ICG-malemide was conjugated with Cys to form ICG-Cys for use as a control. Single tail vein administrations of 2.5 nmol of ICG-Cys or pHLIP ICG in sterile PBS or PBS with 5% DMSO or 5% Ethanol (vol:vol) (volume of the injection was 100 μl) were given to athymic female nude mice (strain Hsd Athymic Nude-Foxn1nu) ranging in age from 5 to 6 weeks (obtained from Envigo RMS Inc). Mice were under gas anesthesia, and imaging of mouse leg and ear was performed immediately and at different time points (from 5 min till 120 min) after administration of ICG-Cys or pHLIP ICG using the Stryker 1558 AIM clinical imaging system with L10 AIM Light Source, 1588 AIM Camera and a 10 mm scope and a Novadaq imaging system. Five animals per construct were used in the study.

##

## Imaging of Blood Vessels in Pigs

Pigs (50 kg) received pHLIP ICG dissolved in PBS or PBS containing 5% Ethanol. Three doses of pHLIP ICG were administered (dose level 1 is 0.052 mg/kg of pHLIP ICG, dose level 2 is an additional administration of 0.12 mg/kg of pHLIP ICG and dose level 3 is an additional administration of 0.24 mg/kg of pHLIP ICG). Each dose was injected intravenously in one bolus followed by a saline flush. Endoscopical and exoscopical imaging was performed at the same time and up to 2 hrs after pHLIP ICG administration. Endoscopic imaging was carried out using the Stryker 1688 system with the L11 light source (808 nm excitation) and adjustable gain. A 10 mm 30-degree Stryker AIM laparoscope was used for imaging of animal internal cavities. Open field imaging was performed using the Stryker SPY-PHI system (805 nm excitation) and fixed gain. Images and video were recorded on a Stryker Connected OR Hub device. The 1688 system has three infrared imaging modes: Overlay, Contrast, and ENV. The overlay mode captures an NIR frame and a white light frame and combines the two to allow surgeons to have the best fluorescence reference (green) while still operating. The contrast mode provides the highest signal-to-noise ratio for the fluorescence signal and does not contain any data from the white light frame. The ENV mode contains the white light frame data in a black/white mode and overlays the fluorescence signal in green to provide a hybrid mode that allows a higher signal-to-noise ratio view of the fluorescence image while still retaining enough of a view of the surrounding tissue to allow the surgeon to continue to operate. The SPY-PHI system has three infrared imaging modes: Overlay, Contrast, and CSF (color-segmented fluorescence). CSF mode is a type of heat map where the fluorescence signal is mapped to color according to intensity. The contrast mode is noticeably more sensitive to viewers than the Overlay or CSF modes for weaker signals.

## Biodistribution and Kinetics

BALB/cAcNHsd mice ranging in age from 5 to 6 weeks obtained from Envigo RMS, Inc. were used in the study. Mouse mammary 4T1 cancer cells were subcutaneously implanted in the right flank (8 x 10^5^ cells/0.1 mL/flank) of adult female or male mice. The triple-negative 4T1 tumor model closely mimics stage IV of human breast cancer. When tumors reached 5­6 mm in diameter, single tail vein injections of 0.5 mg/kg of pHLIP ICG in sterile PBS, or PBS with 5% DMSO or 5% Ethanol (vol:vol) (volume of the injection was 100 μl) were performed. Animals were euthanized 5 min, 1, 2, 4, 6, 16, 26 and 48 hrs after pHLIP ICG administration. Several animals were used for each time point plus seven control animals (no pHLIP ICG administration). Tumor, muscles, skin, heart, lungs, liver, spleen, kidneys, brain, pancreas, bone, stomach, small and large intestines were collected, and imaged immediately after necropsy. After imaging, the organs were weighed and fast frozen in liquid nitrogen. The *ex vivo* fluorescent imaging of organs was performed using Stryker 1588 AIM endoscopic system with L10 AIM Light Source (808 nm excitation and collection of light in the range of approximately of 815 to 850 nm), 1588 AIM Camera using a 10 mm scope. The lens was fixed 4.3 cm away from the surface of the organs, within an enclosed (light-protected) area. The imaging of each organ was performed at three different settings. All conditions were kept constant during the imaging of all organs. The digital images of organs were processed using our program written in Python to determine the average level of the signal. The background signal was determined by introducing an intensity threshold. All the pixels with intensity above the set threshold were counted and the average intensity per pixel was calculated. For organs for which the highest illumination light intensity produced a saturated image, the medium illumination light intensity images were analyzed, then converted to a comparison using experimentally established conversion curves.

The fluorescence signals in organs and tissue were also measured in the tissue/organ homogenates and compared with the signals from the control tissue/organ homogenates (collected from control mice) mixed with known amounts of pHLIP ICG. Samples of about 100 mg of tissue were homogenized with 2.5x (about 250 µL) volumes of DMSO using BioMasher II disposable homogenizers (DiagnoCine, LLC). 30 µl of each homogenate was placed into a 384-well plate and imaged using an Odyssey IR scanner (Li-Cor Biosciences). Tissue homogenates of control mice mixed with known concentrations of pHLIP ICG were used to establish the calibration curve.

##

## Imaging Tumors in Different Tumor Models

Targeting of murine and human tumors was shown in 8 different tumor models in athymic female nude mice (strain Hsd Athymic Nude-Foxn1nu) ranging in age from 5 to 6 weeks (obtained from Envigo RMS, Inc). The following tumors were established by subcutaneous injection of 1 x 10^6^ cells/0.1 ml/flank in flanks of athymic nude mice: HeLa (humans cervical adenocarcinoma), M4A4 (human epithelial carcinoma), 4T1 (murine breast tumor), A549 (human lung carcinoma), LLC (murine Lewis Lung carcinoma), UM-UC3 (human urinary bladder cancer), and 4 x 10^6^ cells/0.1 ml/flank of LNCaP tumor (human prostate cancer). Human MDA-MB-231 (breast adenocarcinoma) tumors were established by injections of 1 x 10^6^ cells/0.05 ml in the mammary fat pad. Tumors reached different sizes (from very small (1­−2 mm in diameter) to large (8­−12 mm in diameter), and 100 μl of tail vein injections of 0.5 mg/kg of pHLIP ICG in sterile PBS or PBS containing either 5% of DMSO or 5% of Ethanol were performed. Imaging was carried out 24 hours after pHLIP ICG administration. White light and NIR whole-body imaging were performed while the animal was under gas (isoflurane) anesthesia using a Stryker 1558 AIM clinical imaging system with L10 AIM Light Source, 1588 AIM Camera and a 10 mm scope. Next, the skin was removed from the tumor side and whole-body imaging of live animals was performed with the skin removed from the tumor side. Finally, surgery was carried out under fluorescence-guidance to remove tumor and to image the tumor bed using the Stryker SPY-PHI handheld clinical imaging system.

**Histopathology**

Tumors with surrounding muscle were frozen in tissue-tek OCT compound using liquid nitrogen and stored at -80°C until sectioned using a cryostat at -25°C (Thermo Scientific HM525 NX) at a 5 µm thickness. The tumor slides were fixed in 4% formaldehyde and stained with hematoxylin and eosin (H&E) (Thermo Fisher Scientific and Poly Scientific R & D Corp). Some sections were covered with a drop of mounting medium (Permount, Fisher Scientific) and then a cover slide was placed over the medium. Stained and non-stained sections were imaged using an Odyssey IR scanner (Li-Cor Biosciences), Styker imaging system, and inverted microscope "Invitrogen EVOS FL Auto 2" using 4x and 10x objectives. Tiled HE images were made using "Invitrogen EVOS FL Auto 2.0 Imaging System" software.

**TABLES**

**Table SI,1**. Qualification of GLP pHLIP ICG verification batch #1912127 manufactured by CordenPharma, GmbH.

| **Characteristic** | **Test Method** | **Specification** | **Results** |
| --- | --- | --- | --- |
| **Appearance** | Visual | Green powder | conforms |
| **Identity** | LC-MS | 1382.9 [M+3H]^3+^/3 ± 1 amu | conforms |
| **Identity** | HPLC | Elution peak with 5% deviation | conforms |
| **Purity, (*p*)** | HPLC | >98.5% | 98.6% |
| **Impurities** | HPLC | Total Imp.: NMT 1.5% | 1.4% |
| **Acetic Acid Content** | IC | ≤ 5% | 4.4% |
| **TFA Content** | IC | Determine and report | 0.01% |
| **Photo-Assay** | NIR | $0.8\cdot p\cdot k$ ≤ OD ≤ $1.11\cdot p\cdot k$ | 0.85 |
| **Residual solvents** | GC | Determine and report | DMF < 100 ppm  ACN < 50 ppm  i-hexane < 50 ppm  MTBE < 50 ppm  DCM < 50 ppm |
| **Water content** | KF | Determine and report | 1.55% |
| **Peptide Content (*k*)** | CHN | Determine and report | N (total): 12.6%  Peptide content: 91% |
| **Cation Content, NH_4_^+^** | IC | Determine and report | 0.79%  (correlates to 0.6% N) |
| **Cation Content, Na^+^** | IC | Determine and report | <0.01% |
| **Peptide Content/Amino Acid Composition** | AAA | Conforms to theory | 87.6% |
| **Peptide Sequence** | MS/MS | Conforms to theory | Conforms to theory, beside AA 1 and 2 |

LC-MS - liquid chromatography - mass spectrometry; HPLC - high performance liquid chromatography; IC – ion chromatography; GC – gas chromatography; KF - Karl-Fischer titration; CHN – carbon, hydrogen, nitrogen element analysis; AAA – amino acid analysis with St.D. <10%; MS/MS – tandem mass spectrometry.

**Table SI,2**. Concentration of pHLIP ICG (µg/mL) in blood of mice and dogs at different time points after single i.v. administration of 12.3 mg/kg of pHLIP ICG to mice and 0.064 mg/kg of pHLIP ICG to dogs.

| **Time post-dose, hours** | **Concentration of pHLIP** **ICG in dog blood, µg/mL** | **Concentration of pHLIP ICG in mice blood, µg/mL** |
| --- | --- | --- |
| 0.08  0.25  0.5  1  2  4  6  8  12  16 | 1.347 ± 0.092  1.230 ± 0.082  1.123 ± 0.089  0.882 ± 0.099  0.749 ± 0.065  0.432 ± 0.056  0.319 ± 0.041  0.207 ± 0.032  0.118 ± 0.008  - | 275.4 ± 7.5  251.5 ± 13.9  239.8 ± 6.3  217.0 ± 8.9  144.0 ± 13.1  102.6 ± 4.8  68.7 ± 1.3  58.8 ± 14.0  -  14.6 ± 2.6 |

**Table SI,3**. Fluorescence intensity (a.u.) obtained by *ex vivo* imaging of organs collected at different time points after i.v. administration of pHLIP ICG into female Balb/C mice bearing 4T1 tumors in right flank.

| **Organ/Time** | **5 min** | **1 hr** | **2 hrs** | **4 hrs** | **6 hrs** | **16 hrs** | **26 hrs** | **48 hrs** |
| --- | --- | --- | --- | --- | --- | --- | --- | --- |
| **Tumor** | 46.3  37.2  32.4  33.4  31.7 | 92.0  117.6  73.8  61.1  157.9 | 119.7  145.3  151.9  121.8  131.1 | 205.3  219.5  191.8  206.4  233.0  111.6  166.6  174.4 | 193.8  169.3  250.5  172.1  207.3 | 208.2  226.8  233.9  214.5  230.6 | 173.3  208.2  182.1  174.8  174.4  215.3  197.1  136.3 | 135.5  167.0  152.0 |
| **Liver** | 380.8  393.6  469.0  450.4  405.4 | 541.2  558.6  519.7  520.5  525.1 | 560.0  568.2  555.7  523.6  505.3 | 532.7  569.4  541.0  539.5  517.6  540.6  553.0  593.2 | 517.1  560.7  489.1  483.9  584.9 | 382.1  386.3  455.1  368.5  410.3 | 213.8  196.9  217.1  214.9  223.0  217.6  209.6  215.9 | 154.4  151.2  143.2 |
| **Kidneys** | 145.9  180.2  165.6  195.7  171.2 | 202.1  202.8  179.8  199.0  184.9 | 175.6  161.0  152.4  154.4  136.8 | 191.7  188.5  175.7  183.6  188.0  178.3  171.0  172.9 | 169.6  169.3  173.3  180.9  176.9 | 154.1  175.2  183.1  160.3  180.4 | 121.4  100.2  108.4  115.2  113.8  117.7  117.0  118.7 | 52.0  71.4  73.9 |
| **Heart** | 174.5  213.0  211.8  191.5  199.6 | 188.4  194.5  170.2  155.5  189.6 | 164.5  154.9  183.9  176.2  158.3 | 191.7  191.6  174.5  182.8  187.5  179.0  181.5  175.9 | 153.6  166.7  155.5  166.5  173.0 | 128.2  115.2  146.4  112.3  143.6 | 67.8  50.1  63.4  61.5  63.6  42.8  52.1  57.9 | 23.3  23.3  23.0 |
| **Lungs** | 214.0  218.3  220.4  215.4  214.8 | 194.4  202.2  192.7  185.8  198.5 | 176.4  163.5  172.4  180.7  191.7 | 187.1  209.3  163.7  196.1  179.0  171.3  159.0  161.4 | 150.6  165.3  141.9  180.4  145.7 | 132.0  115.2  169.5  133.2  156.4 | 54.4  54.5  81.1  53.2  77.6  76.0  69.3  57.9 | 31.5  35.8  35.2 |
| **Brain** | 137.0  153.9  196.2  172.2  175.6 | 126.7  151.5  138.1  136.8  135.4 | 95.4  79.9  96.9  53.8  84.8 | 73.2  78.5  60.0  76.5  81.0  78.8  84.6  92.5 | 50.6  48.7  47.9  55.2  74.3 | 36.1  37.9  35.6  33.7  33.3 | 0  0  0  0  0  0  0  0 | 0  0  0 |
| **Spleen** | 146.1  154.8  168.9  147.9  155.5 | 158.7  162.3  161.1  151.4  155.5 | 134.3  144.2  135.4  144.6  126.2 | 158.3  167.2  143.8  140.0  145.2  144.4  138.4  155.3 | 133.6  160.9  152.7  134.0  159.3 | 102.2  101.4  150.1  92.5  135.3 | 39.9  40.6  50.7  50.8  58.1  60.1  65.4  43.6 | 21.9  22.0  21.9 |
| **Pancreas** | 169.1  176.4  186.6  129.0  104.9 | 163.4  -  140.5  119.1  107.9 | 121.6  96.2  41.3  111.3  74.8 | 101.6  80.2  96.3  77.4  104.7  93.5  89.1  81.9 | 70.7  62.0  87.1  96.3  95.1 | 72.6  49.6  121.2  49.5  58.6 | 46.5  38.4  34.2  35.4  0  0  42.6  28.3 | 0  0  0 |
| **Bone** | 73.8  73.1  88.8  73.4  87.8 | 67.3  92.8  77.0  75.5  57.5 | 40.3  62.5  48.3  96.1  40.4 | 90.1  35.6  55.3  57.5  76.9  80.1  31.2  78.8 | 58.7  73.6  61.2  49.2  90.8 | 76.7  73.4  58.3  64.5  58.2 | 43.1  45.4  42.3  50.2  32.8  68.2  60.2  63.8 | 44.3  55.2  40.5 |
| **Stomach** | 72.8  82.5  104.2  99.6  99.3 | 125.1  81.7  71.8  98.0  118.8 | 49.9  91.1  79.9  98.4  84.3 | 98.5  107.6  109.5  116.4  110.3  136.5  126.1  66.8 | 126.8  129.7  141.6  119.8  116.1 | 103.5  86.3  161.0  92.1  143.6 | 42.2  48.3  71.0  62.2  72.0  52.3  47.9  49.6 | 25.6  36.9  30.7 |
| **Small intestine** | 62.2  45.5  38.4  33.0  33.0 | 78.3  97.2  68.8  62.1  53.0 | 64.3  57.8  31.5  45.4  36.3 | 83.5  54.9  42.1  73.1  70.8  67.0  71.1  44.8 | 40.4  58.8  44.2  68.2  59.5 | 77.6  107.8  78.6  80.9  74.2 | 34.3  43.8  35.8  41.6  33.2  73.0  64.6  61.5 | 0  0  22.7 |
| **Large intestine** | 42.4  35.4  30.0  47.8  29.8 | 49.1  32.5  33.3  27.0  0 | 0  32.2  0  0  0 | 0  39.4  0  0  0  44.0  33.6  23.2 | 32.3  33.4  30.0  39.8  0 | 37.1  42.6  37.8  36.6  45.2 | 0  0  0  0  0  24.9  27.6  0 | 0  0  0 |
| **Skin** | 40.6  1.0  38.0  33.4  27.6 | 32.0  39.4  54.1  38.2  32.1 | 40.9  43.4  0  36.8  39.0 | 66.9  51.0  38.5  39.6  45.1  51.7  59.7  41.5 | 34.5  66.1  56.3  37.3  41.2 | 91.1  60.5  82.5  50.7  98.9 | 0  31.9  0  57.0  36.4  137.2  24.6  29.2 | 37.7  0  0 |
| **Muscle** | 36.5  1.0  1.0  38.6  35.9 | 0  40.8  0  0  0 | 0  0  0  0  0 | 36.2  0  41.7  0  53.0  27.2  25.5  0 | 0  0  0  0  0 | 0  0  0  0  0 | 0  0  42.9  0  0  64.9  0  0 | 0  0  0 |

**Table SI,4.** Mean (and St. D.) of fluorescence intensity values presented in Table SI,3.

| **Organ/Time** | **5 min** | **1 hr** | **2 hrs** | **4 hrs** | **6 hrs** | **16 hrs** | **26 hrs** | **48 hrs** |
| --- | --- | --- | --- | --- | --- | --- | --- | --- |
| **Tumor** | 36.2±6.0 | 100.5±38.5 | 133.9±14.2 | 188.6±38.1 | 198.6±33.0 | 222.8±11.0 | 182.7±24.8 | 151.5±15.7 |
| **Liver** | 419.8±37.9 | 533.0±16.7 | 542.6±26.8 | 548.4±23.5 | 527.1±44.4 | 400.5±34.1 | 213.6±7.7 | 149.6±5.8 |
| **Kidneys** | 171.7±18.4 | 193.7±10.6 | 156.0±14.1 | 181.2±7.8 | 174.0±5.0 | 170.6±12.8 | 114.0±6.8 | 65.8±12.0 |
| **Heart** | 198.1±15.9 | 179.6±16.4 | 167.6±12.2 | 183.1±6.7 | 163.0±8.2 | 129.2±15.7 | 57.4±8.4 | 23.2±0.2 |
| **Lungs** | 216.6±2.7 | 194.7±6.2 | 177.0±10.4 | 178.4±18.0 | 156.8±15.9 | 141.3±21.5 | 65.5±11.7 | 34.1±2.3 |
| **Brain** | 167.0±22.5 | 137.7±8.9 | 82.2±17.4 | 78.1±9.4 | 55.3±11.0 | 35.3±1.8 | 0.0±0.0 | 0.0±0.0 |
| **Spleen** | 154.6±9.0 | 157.8±4.4 | 136.9±7.7 | 149.1±10.1 | 148.1±13.4 | 116.3±25.0 | 51.1±9.5 | 21.9±0.0 |
| **Pancreas** | 153.2±34.7 | 132.7±24.5 | 89.0±32.0 | 90.6±10.1 | 82.2±15.3 | 70.3±30.0 | 28.2±18.2 | 0.0±0.0 |
| **Bone** | 79.4±8.1 | 74.0±13.1 | 57.5±23.4 | 63.2±21.8 | 66.7±16.0 | 66.2±8.5 | 50.8±12.2 | 46.7±7.6 |
| **Stomach** | 91.7±13.4 | 99.1±23.0 | 80.7±18.6 | 108.9±20.7 | 126.8±9.9 | 117.3±33.1 | 55.7±11.3 | 31.0±5.7 |
| **S. Intest.** | 42.4±12.2 | 71.9±16.9 | 47.1±13.9 | 63.4±14.6 | 54.2±11.6 | 83.8±13.6 | 48.5±15.6 | 7.6±13.1 |
| **L. Intest.** | 37.1±7.9 | 28.4±17.9 | 6.4±14.4 | 17.5±19.6 | 27.1±15.6 | 40.5±4.0 | 6.6±12.2 | 0.0±0.0 |
| **Skin** | 27.9±16.4 | 39.1±9.0 | 32.0±18.1 | 49.2±10.1 | 47.1±13.6 | 76.7±20.4 | 39.5±43.7 | 12.6±21.8 |
| **Muscle** | 22.2±20.3 | 8.2±18.2 | 0.0±0.0 | 22.9±20.8 | 0.0±0.0 | 0.0±0.0 | 13.5±25.6 | 0.0±0.0 |

**Figures**

**
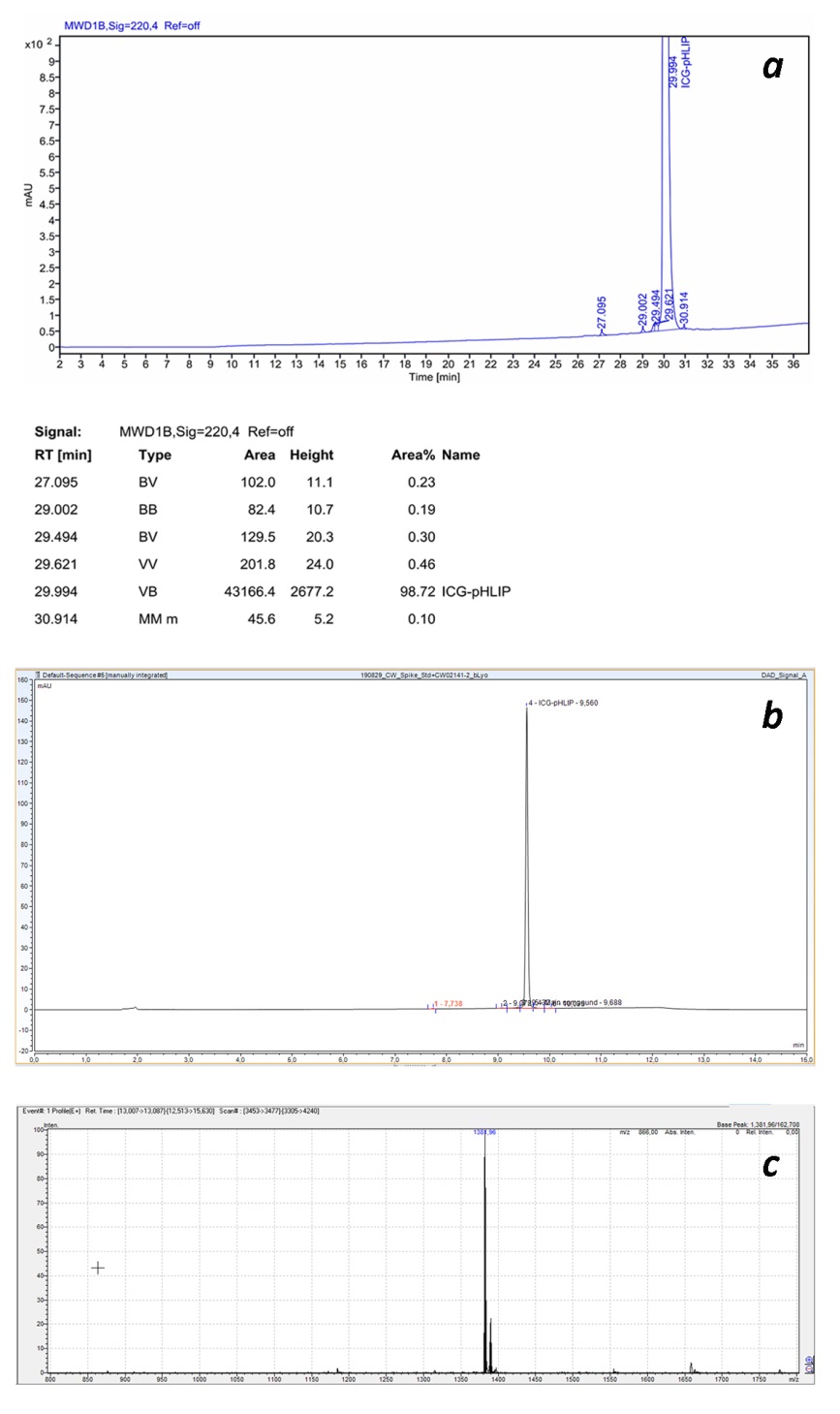
**

**Figure SI,1.** (***a***) HPLC chromatogram of pHLIP ICG used in PoC and toxicology studies. (***b***) The spiking experiment: pHLIP ICG synthesized by CordenPharma according to the optimized protocol was spiked with pHLIP ICG shown on panel ***a*** in ratio of 1:1, dissolved in ACN/water/Tween20 in a ratio of 50:50:0.02 containing 5 mM tri potassium-EDTA and analyzed using the standard analytical method. The HPLC chromatogram provides a single signal, consistent with the compliance of both substances. (***c***) The mass spectrum of pHLIP ICG synthesized by CordenPharma is shown.


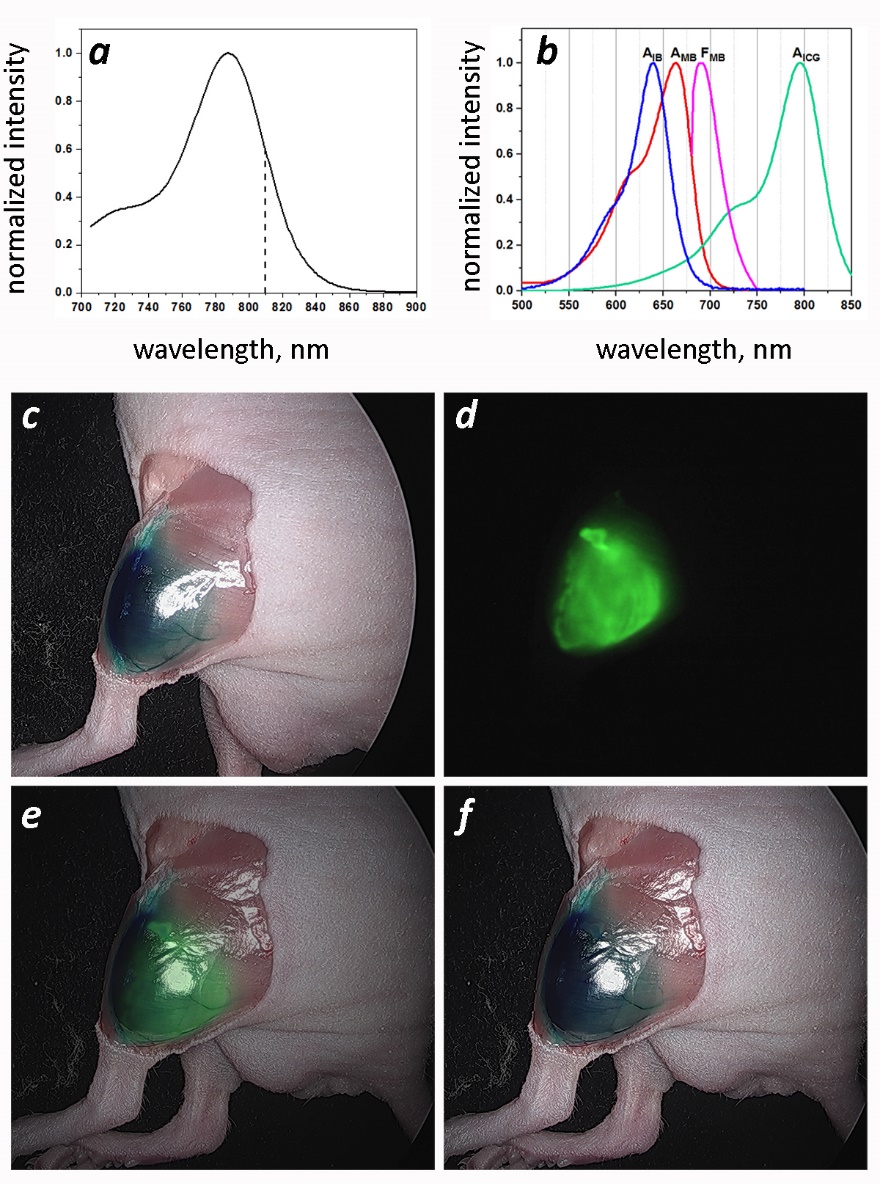


**Figure SI,2**. Normalized absorbance spectrum of pHLIP ICG in methanol (***a***), absorbance and fluorescence spectra of Isosulfan Blue (IB) in water, Methylene Blue (MB) in PBS, and absorbance spectra of pHLIP ICG (ICG) in DMSO (***b***). Color image of mouse received intra-tumoral injection of Isosulfan Blue (***c***). NIRF image of the same mouse followed by intra-tumoral injection of pHLIP ICG (***d***). Overlay of color and NIRF pHLIP ICG images (***e***) and color image of the same mouse after administration of both IB and pHLIP ICG (***f***). The data clearly demonstrate that the administration of pHLIP ICG does not obstruct IB or MB visualization.


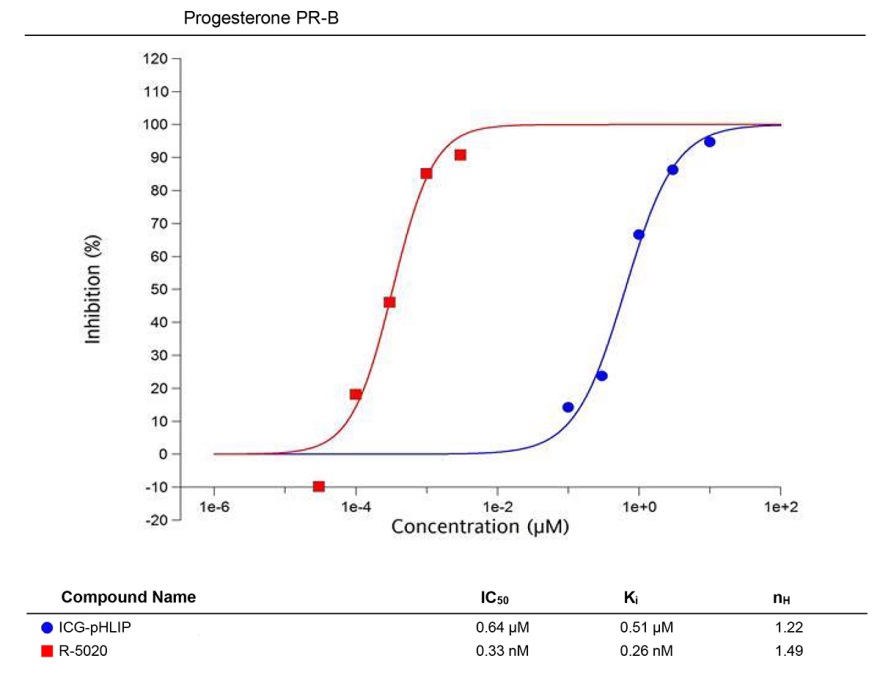


**Figure SI,3**. Inhibition response curves obtained in a radio ligand binding assay after treatment of progesterone B with increasing concentrations of pHLIP ICG (ICG-pHLIP) and R-5020 (a known progesterone B agonist) for 20 hrs. The experiments were performed by Eurofins Panlabs, Inc.


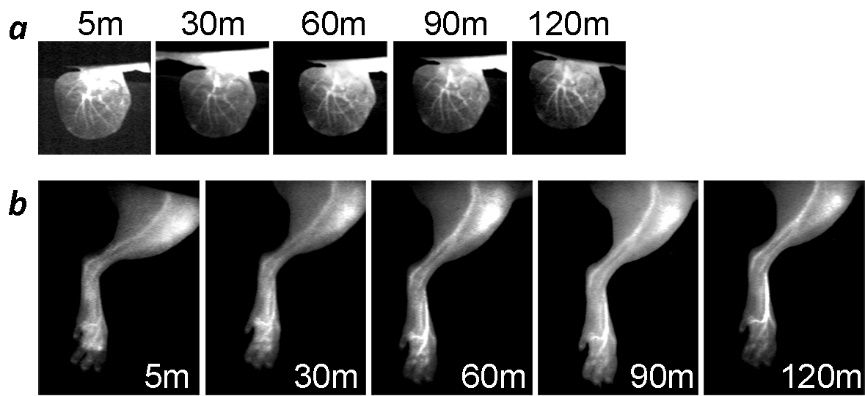


**Figure SI,4**. Fluorescence angiography with pHLIP ICG. Mice under gas anesthesia received a single tail vein injection of 0.5 mg/kg of pHLIP ICG followed by imaging within 2 hrs using a Novadaq imaging system. The signal was recorded from the mouse ear (***a***) and leg (***b***).


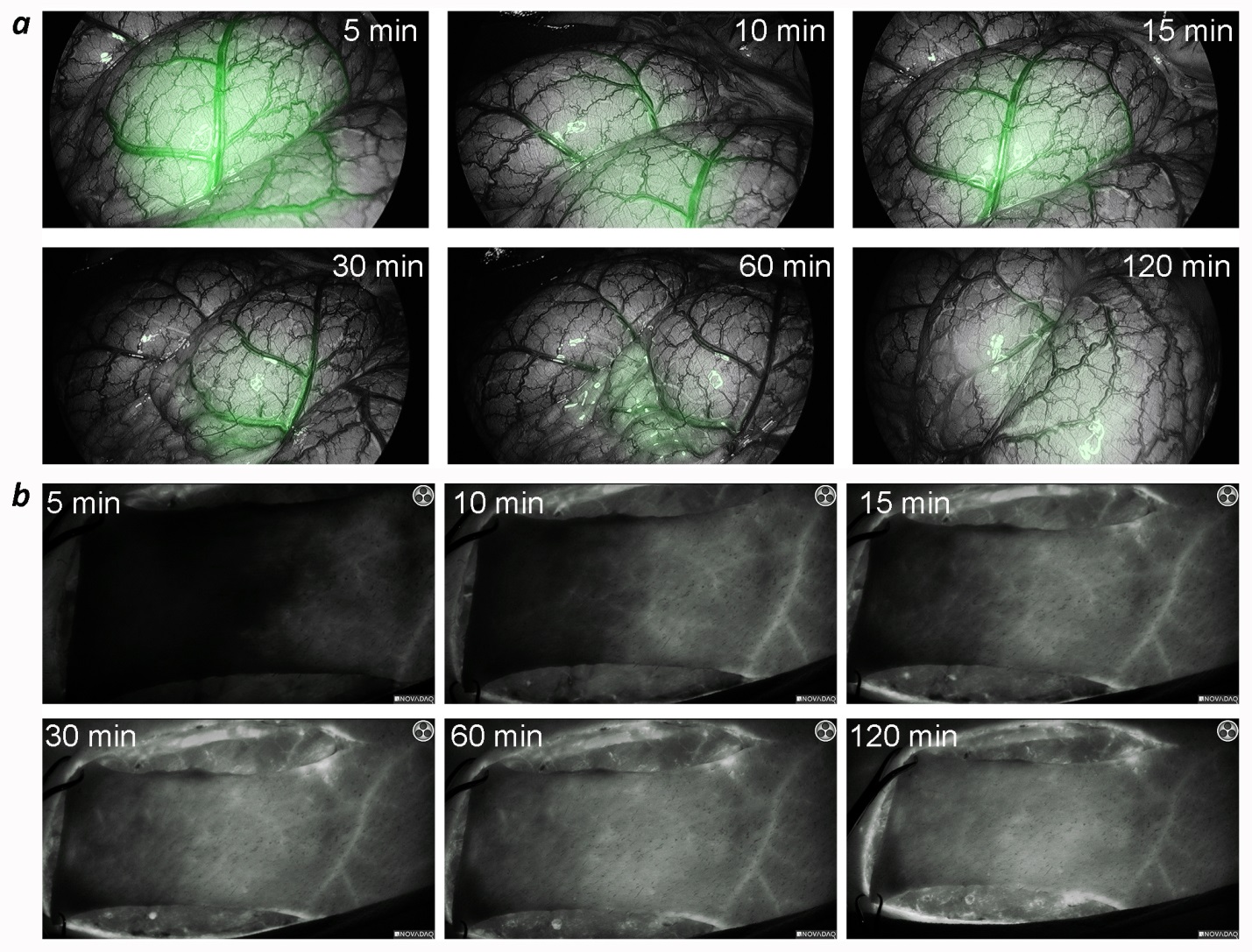


**Figure SI,5**. **Imaging of blood vessels in pigs**. NIR pHLIP ICG fluorescent images of blood vessels at different time points after a single i.v. injection of pHLIP ICG are shown. Imaging inside the pig body cavity was performed using a Stryker system for endoscopic/laparoscopic imaging (***a***). Imaging of a pedicle flap was performed using a Stryker for open field imaging (***b***).


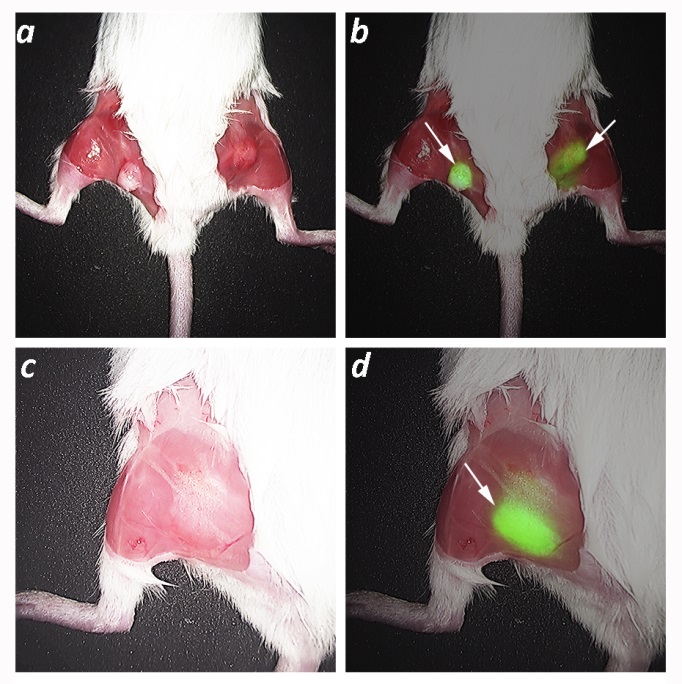


**Figure SI,6.** Photo image of Balb/C mouse bearing 4T1 tumors in both flanks (***a-b***) or in a single flank (***c-d***) with skin removed from the tumor site (***a, c***). Overlays of photo and NIR pHLIP ICG fluorescent images are shown (***b, d***) (tumors are indicated by arrows). pHLIP ICG was administrated as a single i.v. injection (0.5 mg/kg), imaging was performed 24 hrs post-dose while animal was under gas anesthesia and was euthanized immediately after imaging.


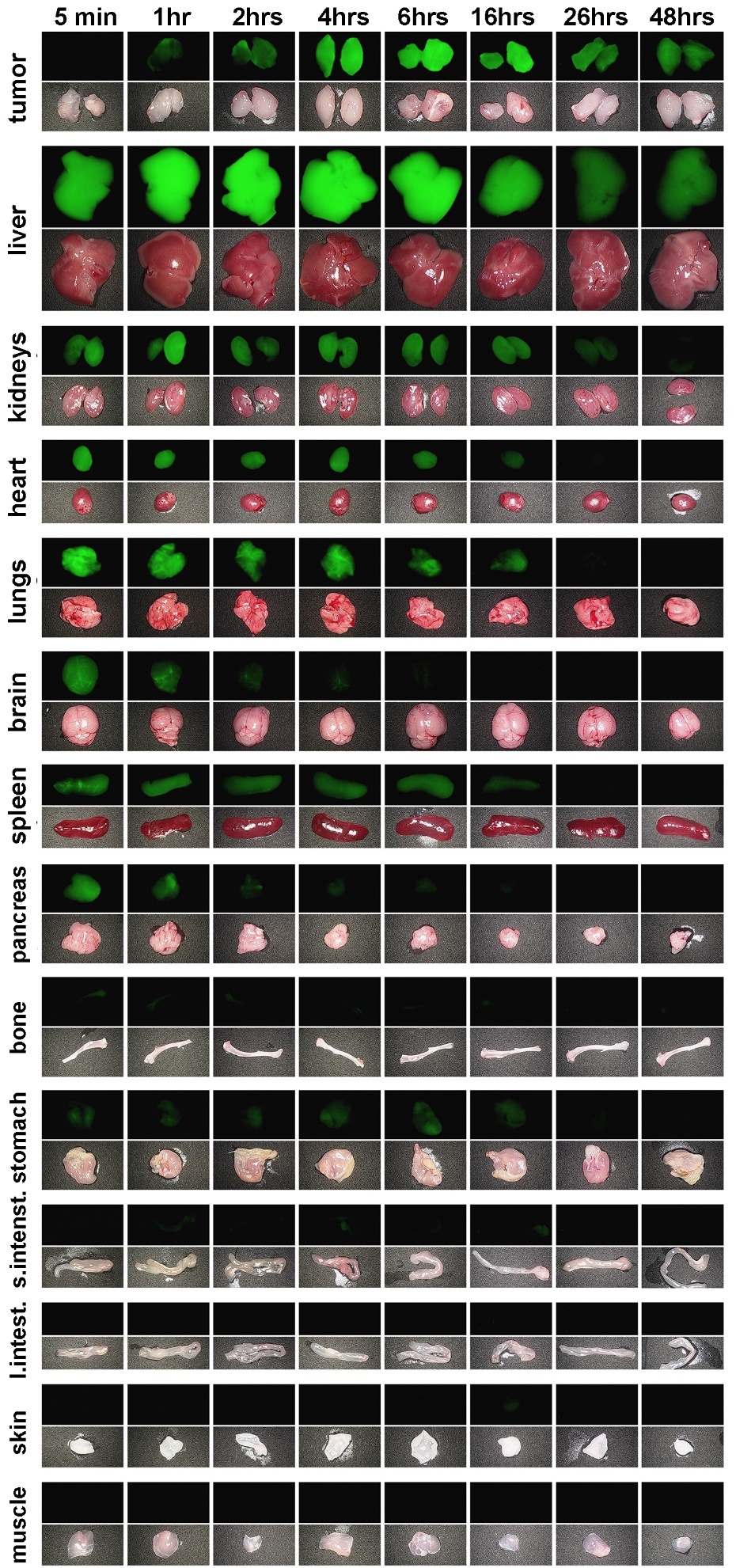


**Figure SI,7.** Representative NIR pHLIP ICG fluorescent and photo images of tumor, major organs and tissues obtained at different time points are shown after a single i.v. administration of pHLIP ICG into BALB/c female mice bearing murine 4T1 breast tumors in right flanks. Tissues and organs were imaged immediately after necropsy. The values of the fluorescence signal obtained for all female mice are presented in Supplementary Tables SI,3 (the NIR fluorescent signals obtained in male mice were similar, data are not shown).


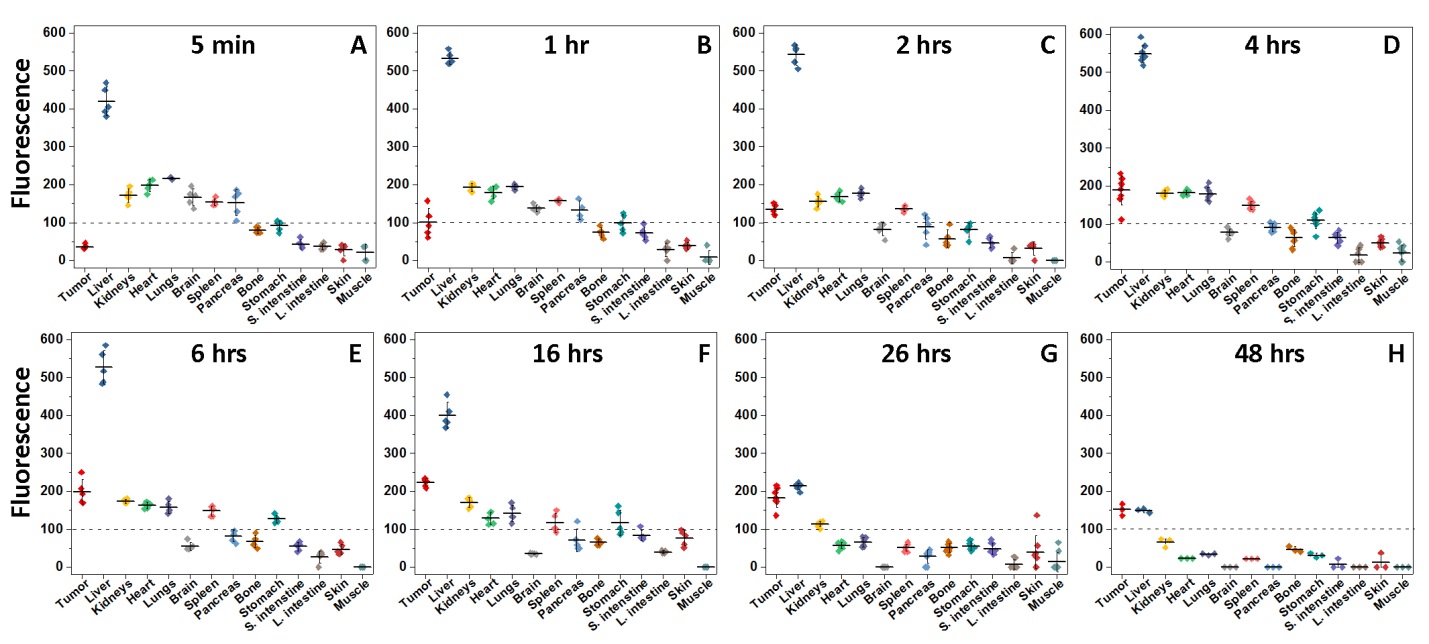


**Figure SI,8.** Tissue/organ mean surface fluorescence calculated from NIR pHLIP ICG fluorescent images for each mouse (the representative images are shown on Figure SI,7 and numbers are given in Table SI,3) and the calculated mean values of fluorescence (numbers are presented in Table SI,4) are shown for different time points post-dose of pHLIP ICG.


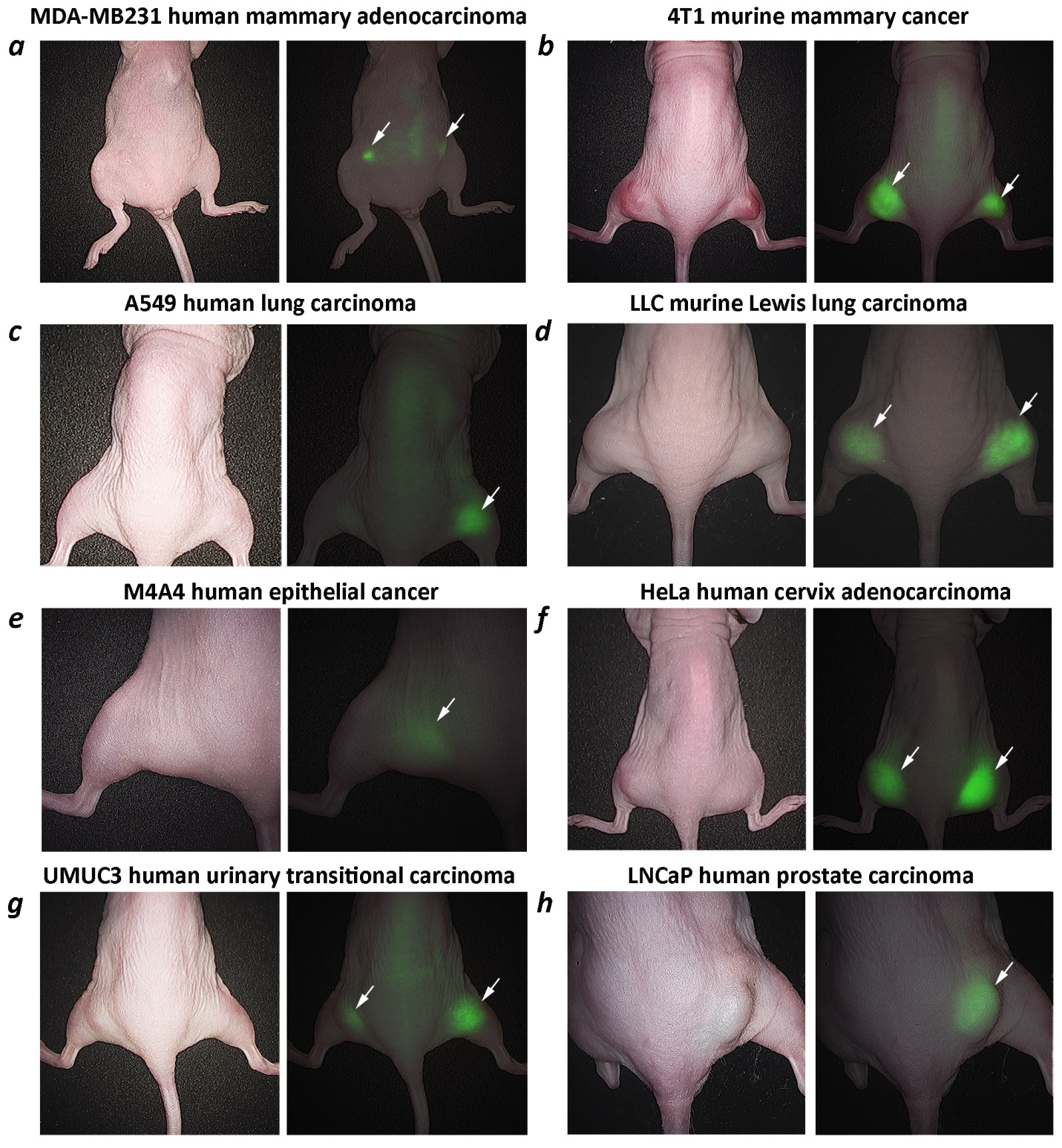


**Figure SI,9.** Photo images and overlay of photo and NIR pHLIP ICG fluorescent images of athymic nude mice bearing human and murine tumors. pHLIP ICG was administrated as a single i.v. injection (0.5 mg/kg), imaging was performed 24 hrs post-dose, while the animal was under gas anesthesia. Tumors are indicated by arrows.


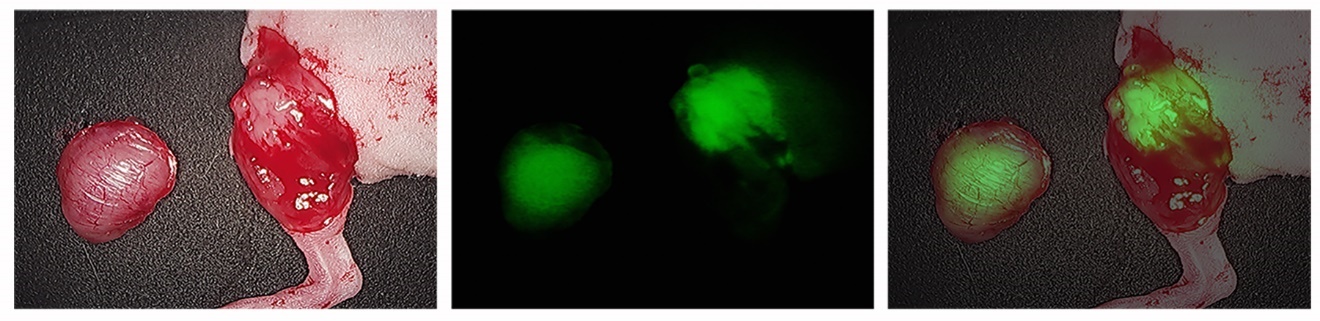


**Figure SI,10.** Removal of tumor and imaging removed tumor and tumor bed (photos, NIR PHLIP ICG fluorescent image and overlay of photo and *ex vivo* NIR PHLIP ICG fluorescent image).

**
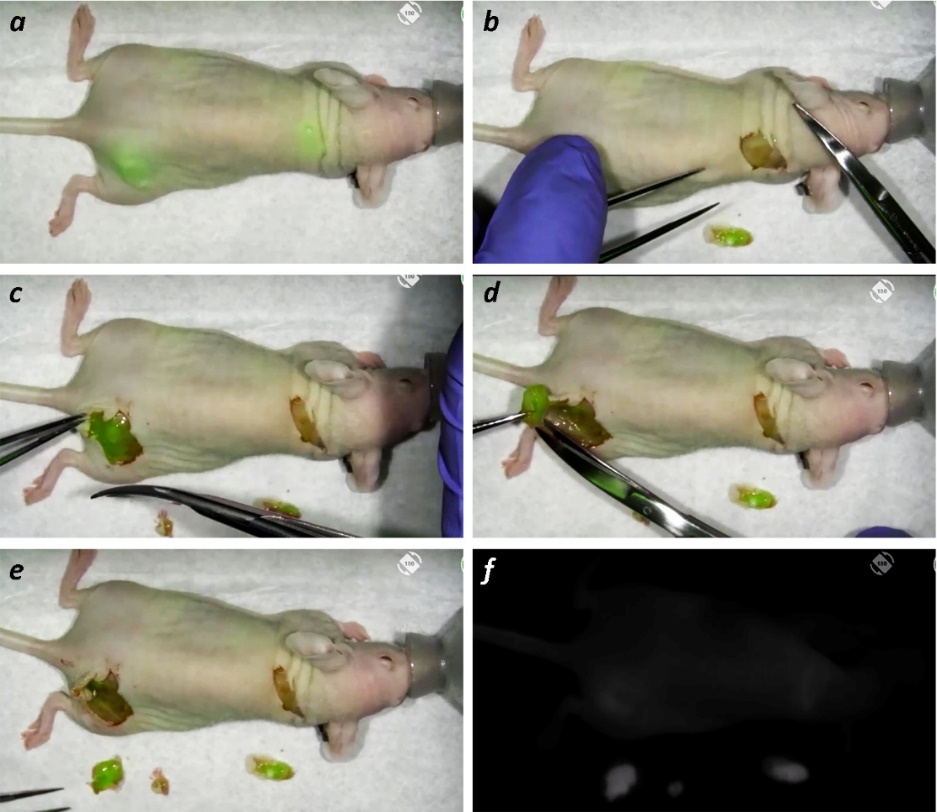
**

**Figure SI,11**. **Surgical removal of tumors under fluorescence guidance**. Representative overlay of color and NIR pHLIP ICG (verification batch #1912127) fluorescent images (***a***-***e***) and NIR fluorescent image (***f***) recorded using a Stryker SPY-PHI handheld device (805 nm excitation) for open field imaging during surgical removal of tumor.
